# Core outcome research measures in anal cancer (CORMAC): protocol for systematic review, qualitative interviews and Delphi survey to develop a core outcome set in anal cancer

**DOI:** 10.1136/bmjopen-2017-018726

**Published:** 2017-11-22

**Authors:** Rebecca Fish, Caroline Sanders, Paula R Williamson, Andrew G Renehan

**Affiliations:** 1 Division of Cancer Sciences, School of Medical Sciences, Faculty of Biology, Medicine and Health, University of Manchester, Manchester, UK; 2 Colorectal and Peritoneal Oncology Centre, Christie NHS Foundation Trust, Manchester, UK; 3 Centre for Primary Care, University of Manchester, Manchester, UK; 4 Department of Biostatistics, University of Liverpool, Liverpool, UK; 5 Medical Research Council North West Hub for Trials Methodology Research, Liverpool, UK

**Keywords:** core outcome set, anal cancer, patient reported outcomes, delphi, radiotherapy

## Abstract

**Introduction:**

The incidence of anal squamous cell carcinoma (ASCC) has increased threefold in the last 30 years. Initial treatment is chemoradiotherapy, associated with short-term and long-term side effects. Future therapy innovations aim to reduce morbidity in treatment of early tumours while maintaining treatment efficacy, and to escalate treatment intensity in locally advanced tumours with acceptable quality of life (QoL). However, all phase III randomised controlled trials to-date have utilised different primary outcomes, which hinders evidence synthesis and presents challenges to the selection of optimal outcomes in future trials. No trial comprehensively assessed long-term side effects and QoL, suggesting outcomes reflecting issues important to patients are under-represented. This project aims to determine the priority outcomes for all stakeholders and reach agreement on a standardised core set of outcomes to be measured and reported on in all future ASCC trials.

**Methods and analysis:**

A systematic review will identify all outcomes reported in trials and observational studies of chemoradiotherapy as primary treatment for ASCC. Outcomes of importance to patients will be identified through patient interviews. The long list of outcomes generated from the systematic review and interviews will be used to create a two-round Delphi process, including key stakeholders (patients and healthcare professionals). The results of the Delphi will be discussed at a face-to-face consensus meeting. Discussion will focus on outcomes that did not achieve consensus through the Delphi process and conclude with anonymous voting to ratify the final core outcome set (COS).

**Ethics and dissemination:**

The final COS will feed directly into the PersonaLising Anal cancer radioTherapy dOse (PLATO) national anal cancer trials and the Association of coloproctologists of Great Britain and Ireland (ACPGBI) supported national anal cancer database. Utilisation of the COS will increase the relevance of research output to all stakeholders and increase the capacity for data synthesis between trials. This study has ethical approval and is registered with the Core Outcome Measures in Effectiveness Trials (COMET) initiative.

**Trial registration number:**

PROSPERO registration ID: CRD42016036540

**Strengths and limitations of this study**

- A core outcome set will facilitate evidence synthesis in anal cancer and ensure future trials utilise outcomes that are relevant to all stakeholders.
- A comprehensive systematic review will identify all outcomes reported in existing trials and observational studies.
- Semi-structured interviews with patients will ensure that outcomes that are important to patients are identified.
- The consensus phase, constituting a Delphi process and face-to-face consensus meeting, includes international professional and patient participation.
- This project will determine which outcomes to measure, but further work will be necessary to agree and recommend a single measurement instrument or definition for each of the outcomes in the core outcome set.

## Introduction

Anal squamous cell carcinoma (ASCC) is no longer an uncommon malignancy. Since the mid-1980s, incidence rates have increased threefold in the UK^1^ with 1247 new cases registered in England in 2012 (approximately 1.5 per 100 000 population). Incidence rates are also increasing in other European populations and in the USA.^2^ Treatment of anal cancer is complex. Initial treatment is chemoradiotherapy, but radical salvage surgery is considered for local relapse which occurs in approximately 20% of cases. Overall, treatment is associated with considerable short-term and long-term side effects. Five-year crude survival is approximately 55%, therefore, there are many long-term survivors living with treatment-related side effects. Future therapy innovations aim to reduce morbidity in early tumours yet maintain treatment efficacy, while escalating treatment intensity with acceptable quality of life (QoL) in locally advanced tumours.

There have been six phase III randomised trials and multiple observational studies of interventions for primary treatment in ASCC, which provide the evidence base for current clinical practice guidelines. Each phase III trial reported different primary outcomes^3^ including local failure rate^4^; locoregional recurrence rate^5^; disease-free survival^6^; colostomy-free survival^7^; complete response^8^ and complete pathological response.^9^ Furthermore, no trial to-date comprehensively assessed long-term side effects and QoL, suggesting outcomes reflecting issues that may be important to patients are under-represented.

Outcome reporting bias generated by selective reporting of subsets of measured outcome variables is demonstrated in up to 62% of published studies^10^ and affects the conclusions in systematic reviews.^11^ Outcomes reaching statistical significance have higher odds of being reported compared with non-significant outcomes,^12^ and harms outcomes reporting is particularly deficient.^13^

Outcome heterogeneity and reporting bias reduce the potential for evidence synthesis, which combined with the narrow scope of reported outcomes presents a significant obstacle to providing healthcare professionals (HCPs) and patients with meaningful information on which to base decisions about treatment. Both these issues may be addressed through the development and use of an agreed standardised collection of outcomes, known as a core outcome set (COS), which should be measured and reported, as a minimum, in all studies and trials for a specific clinical area.^14^ Currently, there is no COS for trials of treatment in patients with ASCC.

There is no agreed gold standard method for COS development. The COMET Initiative (Core Outcome Measures in Effectiveness Trials) is an organisation which aims to facilitate and promote development and use of COSs.^14^ They recommend that COS development utilises rigorous consensus methods which involve all stakeholders, including patients, an approach also advocated by the OMERACT (Outcome Measures in Rheumatology) group.^15^ Here, we will develop a COS for trials in patients with ASCC utilising a recognised stepwise process of information gathering followed by consensus techniques involving all key stakeholder groups.

### Aim

The aim of the project is to determine the priority outcomes for all stakeholders and reach agreement on a standardised COS to be measured and reported in all future trials in patients with ASCC.

### Scope

The scope of the COS to be developed has been defined according to the criteria recommended by COMET.^16^

#### Health condition

Squamous cell carcinoma of the anus/anal canal.

#### Population

Adults >18 years of age.

#### Types of interventions

Primary treatment with radiotherapy with or without concurrent chemotherapy.

#### Setting

Later phase trials that will inform clinical decision making.

## Methods and analysis

Taking into consideration the work of OMERACT and COMET, we selected a mixed-methods approach for COS development. Development will involve four packages of work over two phases. Phase 1 comprises information gathering, employing literature review and qualitative methods of patient consultation. Phase 2 comprises a process of consolidation and consensus employing a Delphi process and structured group discussion involving all stakeholder groups.

### Project oversight

A study advisory group (SAG) has been assembled to oversee the project. Members include oncologists with leading roles in past and current anal cancer clinical trials, a colorectal surgeon, an anal cancer specialist nurse, a COS methodological expert, a qualitative methodology expert and a patient representative.

### Phase 1: information gathering

The aim of the information gathering stage is to generate a comprehensive list of all outcomes relating to the initial treatment of patients with ASCC using chemoradiotherapy. The primary list will be generated by extracting outcomes from the published literature on the subject through a systematic review (WP1). The published literature will be assumed to represent the views of HCPs and trialists. The primary list will be supplemented with any additional outcomes that are identified through a series of semi-structured interviews with individuals who have, or have had anal cancer (WP2).

#### WP1: systematic review

##### Research question

Which outcomes are in use in the published literature on initial treatment of patients with ASCC using radiotherapy with or without concurrent chemotherapy?

##### Method

A systematic review of the literature will be performed to identify a comprehensive list of all outcomes in use in trials and observational studies in patients with ASCC undergoing initial treatments. The full protocol, including search strategy and study selection criteria, is available online via the PROSPERO database.^17^

#### WP2: patient consultation

##### Research question

What are the outcomes patients with anal cancer regard as potentially important following treatment?

##### Method

##### Inclusion criteria

Types of participant

- Adults >18 years of age.
- Patients who have completed or are receiving initial treatment for ASCC.
- Able to participate in an interview in the English language.

Types of pathology

- Anal canal or anal canal and margin cancer of the following histological subtypes that collectively make up the entity of ASCC: squamous cell, basaloid, basosquamous, cloacogenic and transitional cell tumours.

Types of intervention

- External (non-contact) radiotherapy with or without concurrent chemotherapy as initial treatment with curative intent for anal cancer.

##### Exclusion criteria

Types of participant

- Unable to give informed consent.
- Too unwell to comfortably participate in an interview lasting approximately 30–60 min.

Types of pathology

- Anal intraepithelial neoplasia only.
- Anal tumours of histological type other than SCC, including adenocarcinoma, melanoma and other rare tumours.

Types of intervention

- Treatment for anal cancer with purely palliative intent.
- Salvage surgery for anal cancer following primary chemoradiotherapy.
- Any non-radiotherapy initial treatment for anal cancer.

##### Sampling

The majority of participants will be purposively drawn from the prospectively maintained database of patients with anal cancer at the Christie NHS Foundation Trust. We will use existing networks of cancer survivors via national support groups in order to invite participants. A purposive approach to sampling has been selected with the aim of maximising diversity within the study participants. Criteria have been selected to ensure that subsets within the study population that may express contrasting views and experiences are represented. These criteria for difference will be used to populate a sampling matrix (table 1).

**Table 1:** Sampling matrix for purposing sampling of participants in WP2

Key criteria	Target number of participants
Age at diagnosis	
18–30	3–4
31–65	10–12
65+	3–4
Treatment stage	
Undergoing primary treatment	5–7
Completed primary treatment <5 years ago	5–7
Completed primary treatment >5 years ago	5–7
Stoma	
Current stoma or previous stoma	2–4
Gender	
Male	6–8
Female	6–8
Sexuality	
MSM	2–4
HIV status	
HIV positive	2–4
Target total	20

The ‘target total’ refers to the total number of participants but it is not the sum of the individual criteria because many participants will fall into several categories, for example, a male patient with a stoma who completed treatment >5 years ago.

MSM, men who have sex with men.

The key criteria for identifying difference will include:

- Age at diagnosis
- Treatment stage
- HIV status
- Sexuality (specifically men who have sex with men or MSM)
- Gender

In order to ensure inclusion of minority groups which can be hard to reach (eg, MSM), snowball sampling will be used by asking participants to suggest contacts known to them who may be willing to participate. This is a common technique used for researching sensitive topics and for gaining access to hard to reach populations.^18^

##### Sample size

We will conduct up to 30 interviews and final sample size will be contingent on iterative analysis to achieve ‘saturation’ in terms of identifying recurring themes in analysis of the data as described by Francis.^19^

##### Consent

Individuals who do not have capacity to give informed consent will not be included in the study, and any participant who is deemed to have lost capacity to give consent during the study will be withdrawn from the study. Information for potential participants will be provided verbally and in the approved information sheets. It will be stressed that the individual is under no obligation to take part and they are free to withdraw at any time without affecting their medical care.

##### Interview location

Interviews will take place at a time and place convenient to the participant. Choices of location with include:

1. A clinic room at the Christie NHS Foundation Trust
2. A room at the University of Manchester
3. The participant’s home
4. Via telephone

Participants will be reimbursed for travel expenses for travelling to and from interview locations.

##### Interview format

Interviews will explore patients’ perceptions, priorities and experiences of living with and having treatment for anal cancer, using a semistructured format. This approach uses open questions to facilitate a patient-led discussion, guided by additional prompts from a pre-prepared topic guide to ensure key areas are covered (see online supplementary file 1). The topic guide may be modified iteratively during the series of patient interviews to ensure inclusion of items that have been raised by earlier participants but not included in the topic guide are covered in subsequent discussions.

10.1136/bmjopen-2017-018726.supp1 Supplementary material 1

##### Data analysis

Interviews will be audio-recorded and transcribed in full. Transcription will be performed by an approved secretarial service. Data will be analysed through thematic analysis by the framework method^20^ using NVivo V.10 software. The data will be indexed and charted to produce a matrix of themes and cases and these will be discussed and agreed by multiple members of the research team (RF and CS). Themes will be derived from issues raised by participants. From this analysis, we will develop a list of outcomes of key importance to survivors of anal cancer. Only members of the project management group will have access to transcripts.

### Phase 2: consolidation and consensus

A meeting of the SAG will be held to discuss and agree on a comprehensive list of outcomes identified from the patient interviews and systematic review. Discussion of the identified outcomes will ensure clear and efficient meanings are given, and that there is no duplication. The long list created from this meeting will be used to create the Delphi survey used in WP3.

#### WP3: Delphi process

A process of iterative surveys (Delphi process) will be undertaken involving the two key stakeholder groups (patients and HCPs including clinician trialists) adhering to the standards recommended by the COMET Minimum Standards In COS Development project (P Williamson, personal communication). Questionnaires are administered in two sequential rounds, with anonymised feedback of the results of the previous round provided to participants before completion of the subsequent round. This process is intended to achieve consensus among participants by minimising the potential for bias towards the opinions of those who are more outspoken or whose views might be perceived as superior. The aim of the Delphi process is to move towards consensus among stakeholders over which outcomes from the long list generated in phase one should be considered for inclusion in the final COS.

##### Research question

Which outcomes do patients and HCPs think should be included in a core outcome set for trials of patients with ASCC?

##### Method

##### Participants

Participants will be recruited from the two key stakeholder groups: patients and HCPs. Clinicians involved in clinical trials will form a subgroup within the HCP stakeholder group.

##### Inclusion criteria

All participants must be adults >18 years of age and able to complete a questionnaire in the English language

##### Patients

Patients who have completed or are receiving initial treatment using chemoradiotherapy for ASCC.

##### Healthcare professionals

All members of the clinical team involved in the management of individuals who have or have had anal cancer, including all members of the multi-disciplinary team (MDT) are eligible to participate. This will include:

- Clinical oncologists
- Radiologists
- Radiographers
- Pathologists
- Specialist nurses
- Colorectal surgeons
- Stoma nurses
- Gastroenterologists
- Radiophysicists

##### Sampling

##### Patients

All UK centres offering radiotherapy-based treatment for patients with ASCC will be invited to become participant identification centres (PICs). Each PIC will be asked to nominate a member of the clinical team (likely a research nurse or clinical nurse specialist) to identify potential patient participants from clinic lists or patient records. They will then distribute recruitment letters to the identified individuals, either in person during routine follow-up visits, or by post. The recruitment letter will give a full explanation of the Delphi process and instructions of how to contact the research team for more information by email, phone or post. We will ask PICs to display posters advertising the study in appropriate waiting rooms and patient areas. Potential participants contacting the research team will be given details of how to register to take part. The importance of completing all rounds of the Delphi process will be stressed at this stage to try and minimise inter-round attrition.

Links have been established with a number of patient groups internationally (eg, HPV and Anal Cancer Foundation, Pelvic Radiation Disease Association). A named contact at the group will act as a liaison member and will circulate to other members the promotional poster and contact details for the research team. Recruitment posters and email contact for the research team will be disseminated via patient support group websites and via social media sites including twitter.

##### Healthcare professionals

All members of each UK regional anal cancer MDT will be contacted and invited to participate.

The membership of international associations and/or their disease-relevant subgroups will be contacted and invited to participate:

- Association of Coloproctology of Great Britain and Ireland
- European Society of Coloproctology
- European Society for Radiotherapy and Oncology
- American Society of Colon and Rectal Surgeons
- American Society for Radiation Oncology
- Nordic Anal Cancer Group
- Colorectal surgical society of Australia and New Zealand
- Trans-Tasman Radiation Oncology Group

Contacts of the CORMAC SAG will be contacted and invited to participate.

Snowball sampling will be allowed to increase sample size.

##### Trialists

Corresponding authors of the following will be contacted and invited to participate:

- The six phase III randomised trials in anal cancer.
- The working group developing the protocol for the planned international PLATO anal cancer trial.
- Large cohort studies and non-randomised trials published in the last 2 years.
- International Rare Cancers Group.

##### Recruitment

Potential participants will be contacted either by email or post. Correspondence will outline the rationale for the development of a COS and describe the requirements for taking part in the Delphi. In particular, the importance of completing all rounds of the questionnaire will be emphasised in an effort to reduce inter-round dropout.

All participants will be invited to pass on details or the study to any of their own contacts who meet the eligibility requirements (snowball sampling) to increase sample size and reach.

##### Sample size

There are no recommendations for the number of participants to include in a Delphi survey.^21^ We will therefore take a pragmatic approach to sample size and aim to invite all individuals who meet the inclusion criteria as identified by the approach set out above. We will keep a record of the source of all participants and record the number of invited and the number recruited for each stakeholder group. No new participants will be invited after commencement of the round 1 questionnaire.

##### Consent

No explicit consent will be taken for completion of the questionnaire. Consent will be implicit by the process of registering to take part in the Delphi process via the website and by completion and return of questionnaires. It will be clearly stated on the Delphi registration page that registering to participate by submitting their name and email address is indicating their agreement to participate in the Delphi process.

##### Questionnaires

The questionnaire will be built and administered in an online format using the DelphiManager software developed by the COMET group. Participants will be asked to select which of the stakeholder groups (patient; HCP) they belong to prior to commencing the questionnaire.

Further information specific to each stakeholder group will then be gathered:

Patients

- Age
- Months since completion of treatment
- Gender
- Sexuality
- Ethnicity
- Country in which received treatment for ASCC

Healthcare professionals

- Discipline (medical oncologist, specialist nurse, etc).
- Involvement with trials (named author on publication of a trial of chemoradiotherapy in anal cancer; part of working group involved in a trial of chemoradiotherapy in anal cancer; part of working group for development of future trials in anal cancer).
- Country of practice.

Instructions for how to complete the questionnaire will be included at the start of each round. Participants will be asked to rate the importance of each outcome based the scale proposed by the GRADE working group.^22^ This is a 9-point Likert scale, grouped into three categories: 1–3 (limited importance); 4–6 (important but not critical) and 7–9 (critically important).

Within the questionnaire outcomes will be grouped into domains so that similar or related outcomes are viewed together. Each outcome will be described in medical terms and in plain language, with participants able to toggle between versions. The language used will be piloted on patients and HCPs prior to finalising the questionnaire to ensure clarity and consistency of meaning.

Participants will be able to suggest additional outcomes to include in subsequent rounds.

##### Delphi rounds and feedback

Two rounds of the Delphi questionnaire will be undertaken. The spread of scores for each question item should be seen to reduce from round 1 to round 2 as consensus is reached (see definition of consensus in next section).

For all rounds after the first round, participants will be able to review the results from earlier rounds as they rate each outcome. Each participant will be able to see:

1. the score they gave that outcome in earlier rounds,
2. the overall scores given to that outcome by each stakeholder group including their own.

All outcomes from round 1 will be retained for subsequent rounds. The project management group will discuss any additional outcomes proposed by participants in round 1 and decide whether the outcome is included within existing outcomes or should be added as a new outcome for round 2.

##### Attrition between rounds

Although the importance of completion of both rounds of the Delphi survey will be stressed to participants before commencing round 1, it is anticipated that some participants will drop out after each round. Each participant will be ascribed a unique participant number when they sign up to complete round 1 enabling the identification of the attrition rate between rounds. This will allow the identification of participants who have completed both rounds, and analysis of whether participants who drop out before completion of round 2 appear to have views that are different to those who complete the process.

##### Results and analysis

##### Definition of consensus

A clear definition of what constitutes consensus is essential to reduce potential bias in the interpretation of the results in favour of the opinions of the researchers. Consensus can be considered to have been reached if the majority of participants rank an outcome similarly. After the final round, for each stakeholder group, we will assign each outcome to one of three categories:

1. Consensus in
  70% or more respondents within a stakeholder group rate the outcome as critically important (7–9) AND 15% or fewer rate the outcome as limited importance (1–3).
2. Consensus out
  70% or more of respondents within a stakeholder group rate the outcome as limited importance AND 15% or fewer rate the outcome as critically important (7–9).
3. No consensus
  Neither of the above criteria are met.

#### WP4: consensus meetings

##### Research question

Can we ratify a COS for trials in patients with ASCC through a process which involves all stakeholders?

##### Overview

The results of the Delphi process will be discussed at a face-to-face consensus meeting involving an invited sample of Delphi participants from all stakeholder groups. Representatives from secondary stakeholder groups (intended users of the COS including non-clinician trialists; users of the information generated from use of the COS including policy makers guideline developers) will be invited at this stage, in line with the findings of the COMET Minimum Standards in COS development project (P Williamson, personal communication, 2017). At the meeting, we will propose that any outcome categorised as ‘consensus in’ across all stakeholder groups be included in the final COS and any outcome categorised as ‘consensus out’ across all stakeholder groups be excluded. Attendees will electronically vote to accept this proposal or suggest outcomes from this group that warrant further discussion. All other outcomes, including those categorised as ‘consensus in’ or ‘consensus out’ by one or two stakeholder groups, and those categorised as ‘no-consensus’ will then be discussed and further rounds of voting will be used to agree the final COS. If a final COS is not agreed at the end of the first consensus meeting, subsequent meetings will be considered.

##### Recruitment and consent

All participants registering to complete the Delphi process will be additionally offered participation in the consensus meetings (tick box on registration page for Delphi). A sample of participants from both stakeholder groups (patients and HCPs), who have indicated yes to this question and that have completed all rounds of the Delphi process, will be invited to attend the consensus meetings. On the day of the meeting, and prior to commencement of the meeting, patient participants will be asked to confirm their agreement to participate verbally and sign a written consent form.

## Ethics and dissemination

Research ethics committee approval for the interviews in WP2 was granted on 22 December 2015 by the Greater Manchester East research ethics committee. REC reference 15/NW/0971. Research ethics committee approval for the Delphi process in WP3 was granted on 2 December 2016 by the North East – Newcastle & North Tyneside research ethics committee. REC reference 16/NE/0392. HRA approval was granted on 23 December 2016.

The benefits of COS are increasingly recognised by research funding bodies, regulators and journal editors, via the work of the COMET Initiative in promoting COS utilisation. The European Medicines Agency recommends COS use for clinical trials in asthma medicines,^23^ and the UK National Institute for Health Research (NIHR) recommends outcomes from established COS are included in any new trial proposal.^24^

The robust methodology we have proposed for the development of this COS ensures that HCPs, trialists and patients are involved at each stage of development. As a whole, the project is overseen by an advisory group including expert representatives from each of these stakeholder groups. This approach will ensure that outcomes in the final core set accurately represent the priorities of those stakeholders. Additionally, the results from the patient interviews undertaken in WP2 will add substantially to the limited body of published literature available on long-term treatment toxicity following pelvic radiotherapy in patients with anal cancer.

Once the final COS is agreed, additional work is planned to develop a core outcome measurement instrument set, in which a single definition or measurement instrument is recommended for each outcome in the COS. Data gathered in the systematic review undertaken in WP1 will allow identification of existing measurement instruments. Identification of instruments will be followed by an assessment and consensus process as described in the COMET/COSMIN 2016 guideline.^25^

The output from this project will feed directly into the PLATO (PersonaLising Anal cancer radioTherapy dOse) anal cancer trials currently in roll-out,^26^ and into the Association of Coloproctology of Great Britain and Ireland supported national anal cancer audit database. Adoption of the CORMAC COS will help to reduce outcome heterogeneity and therefore increase the quality of information available to HCPs and patients on which to base informed decisions about treatment.

### Study registration

The study is registered with COMET and listed in their online database. http://www.comet-initiative.org/studies/details/781

#### Phase 1 (semistructured interviews)

IRAS ID 183034

CPMS study ID 20368; adopted January 2016

#### Phase 2 (Delphi)

IRAS ID 215791

CPMS Study ID: 33052; adopted February 2017

### Sponsor

The University of Manchester, Christie Building, Oxford Road, Manchester M13 9PL fbmhethics@manchester.ac.uk.

## Supplementary Material

Reviewer comments

Author's manuscript
