# Supplementary material for: Core outcome research measures in anal cancer (CORMAC): protocol for systematic review, qualitative interviews and Delphi survey to develop a core outcome set in anal cancer
Source: BMJ Open. 2017 Nov 22;7(11):e018726. doi: 10.1136/bmjopen-2017-018726 (PMC5719280; doi:10.1136/bmjopen-2017-018726)
Supplement: Supplementary material 1 [file bmjopen-2017-018726supp001.pdf]

CORMAC INTERVIEW TOPIC GUIDE

<table>
<tr><td>Participant No.</td><td></td><td>Interview location:</td><td></td><td>Interview date:</td><td></td></tr>
<tr><td>Date of Birth.</td><td></td><td>Date of diagnosis</td><td></td><td>Date of completion of treatment:</td><td></td></tr>
<tr><td>Gender:</td><td colspan="2">Male □
Female □</td><td>Marital status:</td><td colspan="2">single □
married □
living with partner □</td></tr>
<tr><td>HIV status</td><td colspan="2">Positive □
Negative □
Never tested □</td><td>Sexuality</td><td colspan="2">Homosexual □
Heterosexual □
Bisexual □
Prefer not to answer □</td></tr>
<tr><td>Ethnicity.
(see code sheet)</td><td colspan="2"></td><td>Stoma:</td><td colspan="2">Never □
Reversed □
Temporary □
Permanent □</td></tr>
</table>

Introduction:

- Go over the purpose of the study with participant.
- Check they are still willing to take part.
- Check they are happy for interview to be audio recorded.
- Prompt for and answer any other queries.
- **Ask them to fill in the consent form.**

Interview themes

**1)**	Start with a general question about their experience of having anal cancer<br><br>'I understand you have (had) anal cancer. Can you tell me about that?
**2)**	Ask about their experience of being told of their diagnosis<br><br>*'Could you tell me about how you first found out you had anal cancer?'/ 'If I could take you back to when you first learned about your diagnosis?'*<br><br>Prompt for the questions they most wanted to find answers to on being told their diagnosis<br><br>Ask about the treatment that was offered and how they decided about undergoing treatment<br><br>Prompt for what information they wanted about the treatment they would be receiving, and the factors they considered in deciding on a treatment
**3)**	Ask about the treatment that was offered and how they decided about undergoing treatment<br><br>Prompt for what information they wanted about the treatment they would be receiving, and the factors they considered in deciding on a treatment
**4)**	Ask about the effects that treatment had/ is having<br><br>Prompt for specific areas such as physical, mental, effects on relationships<br><br>Prompt about whether they had to modify their behaviour as a result of treatment<br><br>Ask what they considered to be the worst side effect of treatment
**5**	Ask about the long term or permanent side effects of treatment<br><br>Prompt for specific areas such as physical, mental, effects on relationships<br><br>Prompt about whether they had to modify their behaviour as a result of treatment

	Ask what they considered to be the worst side effect of treatment
**6)**	Ask about concerns for the future, especially those relating to their diagnosis/history of anal cancer
**7)**	Ask if there were any areas they wanted more information about but were unable to find<br><br>Prompt about info leaflets given at time of diagnosis/ treatment
**8)**	Ask whether the explanation of what they should expect from treatment matched their real experience
**9)**	Ask if they can describe what an outcome is in their own words
**10)**	Ask explicitly which outcomes they think it is important to measure
**11)**	Ask whether they think their perspective on what is important has changed over time
