# Supplementary material for: Core outcome research measures in anal cancer (CORMAC): protocol for systematic review, qualitative interviews and Delphi survey to develop a core outcome set in anal cancer
Source: BMJ Open. 2017 Nov 22;7(11):e018726. doi: 10.1136/bmjopen-2017-018726 (PMC5719280; doi:10.1136/bmjopen-2017-018726)
Supplement: Author's manuscript [file bmjopen-2017-018726.draft_revisions.pdf]

BMJ Open is committed to open peer review. As part of this commitment we make the peer review history of every article we publish publicly available.

When an article is published we post the peer reviewers' comments and the authors' responses online. We also post the versions of the paper that were used during peer review. These are the versions that the peer review comments apply to.

The versions of the paper that follow are the versions that were submitted during the peer review process. They are not the versions of record or the final published versions. They should not be cited or distributed as the published version of this manuscript.

BMJ Open is an open access journal and the full, final, typeset and author-corrected version of record of the manuscript is available on our site with no access controls, subscription charges or pay-per-view fees (http://bmjopen.bmj.com).

If you have any questions on BMJ Open's open peer review process please email editorial.bmjopen@bmj.com

BMJ Open

Core Outcome Research Measures in Anal Cancer (CORMAC): protocol for systematic review, qualitative interviews and Delphi survey to develop a core outcome set in anal cancer

Journal:	*BMJ Open*
Manuscript ID	bmjopen-2017-018726
Article Type:	Protocol
Date Submitted by the Author:	18-Jul-2017
Complete List of Authors:	Fish, Rebecca; University of Manchester, Division of Cancer Sciences; The Christie NHS Foundation Trust, Colorectal and Peritoneal Oncology Centre<br>Sanders, Caroline; University of Manchester, Centre for Primary Care<br>Williamson, Paula; University of Liverpool, Department of Biostatistics; Medical Research Council, North West Hub for Trials Methodology Research<br>Renehan, Andrew; University of Manchester, Division of Cancer Sciences; The Christie NHS Foundation Trust, Colorectal and Peritoneal Oncology Centre
**Primary Subject Heading**:	Oncology
Secondary Subject Heading:	Evidence based practice, Health policy, Patient-centred medicine, Qualitative research, Surgery
Keywords:	Core Outcome Set, STATISTICS & RESEARCH METHODS, Anal cancer, Patient reported outcomes, Delphi, RADIOTHERAPY

SCHOLARONE™
Manuscripts

Prepared for submission to BMJ Open July 2017

Core Outcome Research Measures in Anal Cancer (CORMAC): protocol for systematic review, qualitative interviews and Delphi survey to develop a core outcome set in anal cancer

Ms Rebecca Fish[1]; Dr Caroline Sanders[2]; Professor Paula Williamson[3]; Professor Andrew G. Renehan[1,4]

1. Clinical Research Fellow, Division of Cancer Sciences, School of Medical Sciences Faculty of Biology, Medicine and Health, University of Manchester, Vaughan House, Portsmouth Street, Manchester, M13 9GB. rebecca.fish-2@manchester.ac.uk

2. Senior Lecturer in Medical Sociology, Centre for Primary Care, University of Manchester, Williamson Building, 6th Floor, Suite 3, Manchester, M13 9PL. Caroline.sanders@manchester.ac.uk

3. Professor of Medical Statistics, Department of Biostatistics, Block F Waterhouse Building, University of Liverpool, 1-5 Brownlow Street, Liverpool, L69 3GL. P.R.Williamson@liverpool.ac.uk

4. Professor in Cancer Studies and Surgery, Honorary Consultant Peritoneal and Colorectal Cancer Surgeon Peritoneal and Colorectal Oncology Centre, Christie NHS Foundation Trust, 550 Wilmslow Road, Manchester, M20 4BX. andrew.renehan@ics.manchester.ac.uk

Correspondence to:

Dr Rebecca Fish
Clinical Research Fellow,
Division of Cancer Sciences, School of Medical Sciences
Faculty of Biology, Medicine and Health,
University of Manchester,
Vaughan House, Portsmouth Street,
Manchester, M13 9GB.
E-mail: rebecca.fish-2@manchester.ac.uk

Abstract: 299 words; main text: 3994 words; 1 table; 26 references; 1 supplementary file; language: UK English.

For peer review only - http://bmjopen.bmj.com/site/about/guidelines.xhtml

ABSTRACT

Introduction

The incidence of anal squamous cell carcinoma (ASCC) has increased 3-fold in the last 30 years. Initial treatment is chemoradiotherapy, associated with considerable short and long-term side effects. Future therapy innovations aim to reduce morbidity in treatment of early tumours whilst maintaining treatment efficacy, and to escalate treatment intensity in locally advanced tumours with acceptable quality of life (QoL). However, all phase III randomised controlled trials to-date have utilised different primary outcomes, which hinders evidence synthesis and presents challenges to the selection of optimal outcomes in future trials. No trial comprehensively assessed long-term side effects and QoL, suggesting outcomes reflecting issues important to patients are under-represented. This project aims to determine the priority outcomes for all stakeholders and reach agreement on a standardized core set of outcomes to be measured and reported on in all future ASCC trials.

Methods and Analysis

A systematic review will identify all outcomes reported in trials and observational studies of chemoradiotherapy as primary treatment for ASCC. Semi-structured interviews with patients followed by qualitative analysis will identify outcomes of importance to patients supplementing the list generated from the systematic review. The long list of outcomes generated from the systematic review and interviews will be used to create a Delphi process conducted over two rounds including key stakeholders (patients, health care professionals). The results from the Delphi will be discussed at a face-to-face consensus meeting. Discussion will focus on outcomes that did not achieve consensus through the Delphi process and conclude with anonymous voting with predefined criteria for consensus to ratify the final core outcome set (COS).

Ethics and Dissemination

The COS developed will inform future treatment effectiveness trials, for example, the PLATO trial. Utilisation of the COS will increase the relevance of research output to all stakeholders and increase the capacity for data synthesis between trials.

For peer review only - http://bmjopen.bmj.com/site/about/guidelines.xhtml

Prepared for submission to BMJ Open July 2017

STUDY REGISTRATION

The study is registered with COMET and listed in their online database.

http://www.comet-initiative.org/studies/details/781

Systematic review:

PROSPERO registration ID: CPMS20368

Phase 1 (semi-structured interviews)

IRAS ID 183034

CPMS study ID; adopted January 2016

Phase 2 (Delphi)

IRAS ID 215791

CPMS Study ID: 33052; adopted February 2017

SPONSOR

The University of Manchester, Christie Building, Oxford Road, Manchester M13 9PL

fbmhethics@manchester.ac.uk.

For peer review only

STRENGTHS AND LIMITATIONS OF THIS STUDY

- A core outcome set will facilitate evidence synthesis in anal cancer and ensure future trials utilize outcomes that are relevant to all stakeholders.
- A comprehensive systematic review will identify all outcomes reported in existing trials and observational studies
- Semi-structured interviews with patients will ensure that outcomes that are important to patients are identified
- The consensus phase, constituting a Delphi process and face-to-face consensus meeting, includes international professional and patient participation.
- This project will be followed up with work to recommend or develop appropriate measurement instruments for the outcomes selected.

For peer review only

For peer review only - http://bmjopen.bmj.com/site/about/guidelines.xhtml

INTRODUCTION

Anal squamous cell carcinoma (ASCC) is no longer an uncommon malignancy. Since the mid-1980s, incidence rates have increased 3-fold in the UK [1] with 1247 new cases registered in England in 2012 (approximately 1.5 per 100,000 population). Incidence rates are also increasing in other European populations and the in the United States [2]. Treatment of anal cancer is complex. Initial treatment is chemo-radiotherapy, but radical salvage surgery is considered for local relapse which occurs in approximately 20% of cases. Overall, treatment is associated with considerable short- and long-term side effects. Five-year crude survival is approximately 55%, therefore there are many long-term survivors living with treatment-related side effects. Future therapy innovations aim to reduce morbidity in early tumours yet maintain treatment efficacy, whilst escalating treatment intensity with acceptable quality of life (QoL) in locally advanced tumours.

There have been six phase III randomized trials and multiple observational studies of interventions for primary treatment in ASCC, which provide the evidence base for current clinical practice guidelines. Each phase III trial reported different primary outcomes [3] including local failure rate [4]; loco-regional recurrence rate [5]; disease-free survival [6]; colostomy-free survival [7]; complete response [8] and complete pathological response [9]. Furthermore, no trial to-date comprehensively assessed long-term side effects and QoL, suggesting outcomes reflecting issues that may be important to patients are under-represented.

Outcome reporting bias generated by selective reporting of subsets of measured outcome variables is demonstrated in up to 62% of published studies [10] and affects the conclusions in systematic reviews [11]. Outcomes reaching statistical significance have higher odds of being reported compared to non-significant outcomes [12], and harms outcomes reporting is particularly deficient [13].

Outcome heterogeneity and reporting bias reduce the potential for evidence synthesis, which combined with the narrow scope of reported outcomes presents a significant obstacle to

For peer review only

providing health care professionals and patients with meaningful information on which to base decisions about treatment. Both these issues may be addressed through the development and use of an agreed standardized collection of outcomes, known as a core outcome set (COS), which should be measured and reported, as a minimum, in all studies and trials for a specific clinical area [14]. Currently, there is no COS for trials of treatment in patients with ASCC.

There is no agreed gold standard method for COS development. The COMET Initiative (Core Outcome Measures in Effectiveness Trials) is an organization which aims to facilitate and promote development and use of core outcome sets [14]. They recommend that COS development utilises rigorous consensus methods which involve all stakeholders, including patients, an approach also advocated by the OMERACT (Outcome measures in Rheumatology) group [15]. Here, we will develop a COS for trials in patients with ASCC utilising a recognised stepwise process of information gathering followed by consensus techniques involving all key stakeholder groups.

Aim

The aim of the project is to determine the priority outcomes for all stakeholders and reach agreement on a standardized COS to be measured and reported in all future trials in patients with ASCC.

Scope

The scope of the core outcome set to be developed has been defined according to the criteria recommended by COMET [16].

Health condition: Squamous cell carcinoma of the anus/ anal canal (ASCC)

Population: Adults >18 years of age

Types of Interventions: Primary treatment with radiotherapy with or without concurrent chemotherapy

Setting: Later phase trials that will inform clinical decision making

For peer review only - http://bmjopen.bmj.com/site/about/guidelines.xhtml

For peer review only

METHODS AND ANALYSIS

Taking into consideration the work of OMERACT and COMET, we selected a mixed methods approach for COS development. Development will involve four packages of work over two phases. Phase 1 comprises information gathering, employing literature review and qualitative methods of patient consultation. Phase 2 comprises a process of consolidation and consensus employing a Delphi process and structured group discussion involving all stakeholder groups.

Project oversight

A study advisory group (SAG) has been assembled to oversee the project. Members include oncologists with leading roles in past and current anal cancer clinical trials, a colorectal surgeon, an anal cancer specialist nurse, a COS methodological expert, a qualitative methodology expert and a patient representative.

Phase 1: Information gathering

The aim of the information gathering stage is to generate a comprehensive list of all outcomes relating to the initial treatment of patients with ASCC using chemoradiotherapy. The primary list will be generated by extracting outcomes from the published literature on the subject through a systematic review (WP1). The published literature will be assumed to represent the views of health care professionals and trialists. The primary list will be supplemented with any additional outcomes that are identified through a series of semi-structured interviews with individuals who have, or have had anal cancer (WP2).

For peer review only - http://bmjopen.bmj.com/site/about/guidelines.xhtml

WP1: Systematic review

Research question

Which outcomes are in use in the published literature on initial treatment of patients with ASCC using radiotherapy with or without concurrent chemotherapy?

Method

A systematic review of the literature will be performed to identify a comprehensive list of all outcomes in use in trials and observational studies in patients with ASCC undergoing initial treatments. The full protocol, including search strategy and study selection criteria, is available online via the PROSPERO database [17].

WP2: Patient consultation

Research Question

What are the outcomes patients with anal cancer regard as potentially important following treatment?

Methods

Inclusion criteria

Types of participant

- Adults > 18 years of age.
- Patients who have completed or are receiving initial treatment for ASCC
- Able to participate in an interview in the English language

Types of pathology

- Anal canal or anal canal and margin cancer of the following histological sub-types that collectively make up the entity of ASCC: squamous cell, basaloid, baso-squamous, cloacogenic and transitional cell tumours.

For peer review only - http://bmjopen.bmj.com/site/about/guidelines.xhtml

For peer review only

Types of Intervention

- External (non-contact) radiotherapy with or without concurrent chemotherapy as initial treatment with curative intent for anal cancer.

Exclusion criteria

Types of participant

- Unable to give informed consent
- Too unwell to comfortably participate in an interview lasting approximately 30-60 minutes. Types of pathology

Types of pathology

- Anal intra-epithelial neoplasia (AIN) only
- Anal tumours of histological type other than SCC, including adenocarcinoma, melanoma and other rare tumours

Types of intervention

- Treatment for anal cancer with purely palliative intent
- Salvage surgery for anal cancer following primary chemo-radiotherapy
- Any non-radiotherapy initial treatment for anal cancer

Sampling

The majority of participants will be purposively drawn from the prospectively maintained database of patients with anal cancer at The Christie NHS Foundation Trust. We will utilise existing networks of cancer survivors via national support groups in order to invite participants. A purposive approach to sampling has been selected with the aim of maximising diversity within the study participants. Criteria have been selected to ensure that subsets within the study population that may express contrasting views and experiences are represented. These criteria for difference will be used to populate a sampling matrix (Table 1).

Table 1 Sampling matrix for purposing sampling of participants in WP2.

For peer review only - http://bmjopen.bmj.com/site/about/guidelines.xhtml

**Key Criteria**	**Target Number of participants**
**Age at diagnosis**	
18-30	3-4
31-65	10-12
65+	3-4
**Treatment stage**	
Undergoing primary treatment	5-7
Completed primary treatment <5 years ago	5-7
Completed primary treatment >5 years ago	5-7
**Stoma**	
Current stoma or previous stoma	2-4
**Gender**	
Male	6-8
Female	6-8
**Sexuality**	
MSM	2-4
**HIV status**	
HIV positive	2-4
**Target total**	**20**

MSM: Men who have sex with men *Note: The 'Target Total' refers to the total number of participants but it is not the sum of the individual criteria because many participants will fall into several categories e.g. a male patient with a stoma who completed treatment >5 years ago.*

The key criteria for identifying difference will include:

- Age at diagnosis
- Treatment stage
- HIV status
- Sexuality (specifically men who have sex with men or MSM)
- Gender

In order to ensure inclusion of minority groups which can be hard to reach (e.g. MSM), snowball sampling will be used by asking participants to suggest contacts known to them

For peer review only - http://bmjopen.bmj.com/site/about/guidelines.xhtml

who may be willing to participate. This is a common technique used for researching sensitive topics and for gaining access to hard to reach populations [18].

Sample Size

We will conduct up to 30 interviews and final sample size will be contingent on iterative analysis to achieve 'saturation' in terms of identifying recurring themes in analysis of the data as described by Francis [19].

For peer review only

Consent

Individuals who do not have capacity to give informed consent will not be included in the study, and any participant who is deemed to have lost capacity to give consent during the study will be withdrawn from the study. Information for potential participants will be provided verbally and in the approved information sheets. It will be stressed that the individual is under no obligation to take part and they are free to withdraw at any time without affecting their medical care.

Interview location

Interviews will take place at a time and place convenient to the participant. Choices of location with include:

1. A clinic room at the Christie NHS Foundation Trust
2. A room at the University of Manchester
3. The participant’s home
4. Via telephone

Participants will be reimbursed for travel expenses for travelling to and from interview locations.

Interview format

For peer review only - http://bmjopen.bmj.com/site/about/guidelines.xhtml

Interviews will explore patients' perceptions, priorities and experiences of living with and having treatment for anal cancer, using a semi-structured format. This approach uses open questions to facilitate a patient-led discussion, guided by additional prompts from a pre-prepared topic guide to ensure key areas are covered [see supplementary file 1]. The topic guide may be modified iteratively during the series of patient interviews to ensure inclusion of items that have been raised by earlier participants but not included in the topic guide are covered in subsequent discussions.

Data Analysis

Interviews will be audio-recorded and transcribed in full. Transcription will be performed by an approved secretarial service. Data will be analysed through thematic analysis by the Framework method [20] using NVivo 10 software. The data will be indexed and charted to produce a matrix of themes and cases and these will be discussed and agreed by multiple members of the research team (RF & CS). Themes will be derived from issues raised by participants. From this analysis, we will develop a list of outcomes of key importance to survivors of anal cancer. Only members of the project management group will have access to transcripts.

Phase 2: Consolidation and Consensus

A meeting of the study advisory group will be held to discuss and agree on a comprehensive list of outcomes identified from the patient interviews and systematic review. Discussion of the identified outcomes will ensure clear and efficient meanings are given, and that there is no duplication. The long list created from this meeting will be used to create the Delphi survey used in WP3.

WP3: Delphi process

A process of iterative surveys (Delphi process) will be undertaken involving the two key stakeholder groups (patients and health care professionals including clinician trialists)

For peer review only - http://bmjopen.bmj.com/site/about/guidelines.xhtml

For peer review only

adhering to the standards recommended by the COMET Minimum Standards In COS Development project (Paula Williamson, personal communication). Questionnaires are administered in 2 sequential rounds, with anonymised feedback of the results of the previous round provided to participants before completion of the subsequent round. This process is intended to achieve consensus amongst participants by minimising the potential for bias towards the opinions of those who are more outspoken or whose views might be perceived as superior. The aim of the Delphi process is to move towards consensus amongst stakeholders over which outcomes from the long list generated in phase 1 should be considered for inclusion in the final COS.

Research question

Which outcomes do patients and health care professionals think should be included in a core outcome set for trials of patients with ASCC?

Method

Participants

Participants will be recruited from the two key stakeholder groups: patients and health care professionals (HCPs). Clinicians involved in clinical trials will form a subgroup within the HCP stakeholder group.

Inclusion criteria

All participants must be adults > 18 years of age and able to complete a questionnaire in the English language

Patients

- Patients who have completed or are receiving initial treatment using chemoradiotherapy for ASCC.

Health care professionals

For peer review only - http://bmjopen.bmj.com/site/about/guidelines.xhtml

All members of the clinical team involved in the management of individuals who have or have had anal cancer, including all members of the MRT are eligible to participate. This will include:

- Clinical oncologists
- Radiologists
- Radiographers
- Pathologists
- Specialist nurses
- Colorectal surgeons
- Stoma nurses
- Gastroenterologists
- Radio-physicists

Sampling

Patients

All UK centres offering radiotherapy based treatment for patients with ASCC will be invited to become participant identification centres (PICs). Each PIC will be asked to nominate a member of the clinical team (likely a research nurse or clinical nurse specialist) to identify potential patient participants from clinic lists or patient records. They will then distribute recruitment letters to the identified individuals, either in person during routine follow-up visits, or by post. The recruitment letter will give a full explanation of the Delphi process and instructions of how to contact the research team for more information by e-mail, phone or post. We will ask PICs to display posters advertising the study in appropriate waiting rooms and patient areas. Potential participants contacting the research team will be given details of how to register to take part. The importance of completing all rounds of the Delphi process will be stressed at this stage to try and minimise inter-round attrition.

For peer review only - http://bmjopen.bmj.com/site/about/guidelines.xhtml

For peer review only

Links have been established with a number of patient groups internationally (for example, HPV and Anal Cancer Foundation, Pelvic Radiation Disease Association). A named contact at the group will act as a liaison member and will circulate to other members the promotional poster and contact details for the research team. Recruitment posters and e-mail contact for the research team will be disseminated via patient support group websites and via social media sites including twitter.

Health Care Professionals

All members of each UK regional anal cancer MDT will be contacted and invited to participate.

The membership of international associations and/or their disease relevant subgroups will be contacted and invited to participate:

- Association of Coloproctology of Great Britain and Ireland
- European Society of Coloproctology
- European Society for Radiotherapy and Oncology
- American Society of Colon and Rectal Surgeons
- American Society for Radiation Oncology
- Nordic Anal Cancer Group
- Colorectal surgical society of Australia and New Zealand
- Trans-Tasman Radiation Oncology Group

Contacts of the CORMAC study advisory group will be contacted and invited to participate.

Snowball sampling will be allowed to increase sample size.

Trialists

Corresponding authors of the following will be contacted and invited to participate:

- The 6 phase III randomised trials in anal cancer
- The working group developing the protocol for the planned international PLATO anal cancer trial

For peer review only - http://bmjopen.bmj.com/site/about/guidelines.xhtml

- Large cohort studies and non-randomised trials published in the last 2 years.
- International Rare Cancers Group

Recruitment

Potential participants will be contacted either by e-mail or post. Correspondence will outline the rationale for the development of a core outcome set and describe the requirements for taking part in the Delphi. In particular, the importance of completing all rounds of the questionnaire will be emphasised in an effort to reduce inter-round drop-out.

All participants will be invited to pass on details or the study to any of their own contacts who meet the eligibility requirements (snowball sampling) to increase sample size and reach.

Sample size

There are no recommendations for the number of participants to include in a Delphi survey [21]. We will therefore take a pragmatic approach to sample size and aim to invite all individuals who meet the inclusion criteria as identified by the approach set out above. We will keep a record of the source of all participants and record the number of invited and the number recruited for each stakeholder group. No new participants will be invited after commencement of the round 1 questionnaire.

Consent

No explicit consent will be taken for completion of the questionnaire. Consent will be implicit by the process of registering to take part in the Delphi process via the website and by completion and return of questionnaires. It will be clearly stated on the Delphi registration page that registering to participate by submitting their name and e-mail address is indicating their agreement to participate in the Delphi process.

Questionnaires

For peer review only - http://bmjopen.bmj.com/site/about/guidelines.xhtml

The questionnaire will be built and administered in an online format using the DelphiManager software developed by the COMET group. Participants will be asked to select which of the stakeholder groups (patient; HCP) they belong to prior to commencing the questionnaire. Further information specific to each stakeholder group will then be gathered:

Patients

- Age
- Months since completion of treatment
- Gender
- Sexuality
- Ethnicity
- Country in which received treatment for ASCC

Health care professionals

- Discipline (medical oncologist, specialist nurse etc)
- Involvement with trials (named author on publication of a trial of chemoradiotherapy in anal cancer; part of working group involved in a trial of chemoradiotherapy in anal cancer; part of working group for development of future trials in anal cancer
- Country of practice

Instructions for how to complete the questionnaire will be included at the start of each round. Participants will be asked to rate the importance of each outcome based the scale proposed by the GRADE working group [22]. This is a 9 point Likert scale, grouped into 3 categories: 1-3 (limited importance); 4-6 (important but not critical) and 7-9 (critically important).

Within the questionnaire outcomes will be grouped into domains so that similar or related outcomes are viewed together. Each outcome will be described in medical terms and in plain language, with participants able to toggle between versions. The language used will be

For peer review only - http://bmjopen.bmj.com/site/about/guidelines.xhtml

For peer review only

piloted on patients and health care professionals prior to finalising the questionnaire to ensure clarity and consistency of meaning.

Participants will be able to suggest additional outcomes to include in subsequent rounds.

Delphi rounds and feedback

Two rounds of the Delphi questionnaire will be undertaken. The spread of scores for each question item should be seen to reduce from round 1 to round 2 as consensus is reached (see definition of consensus in next section).

For all rounds after the first round, participants will be able to review the results from earlier rounds as they rate each outcome. Each participant will be able to see:

1. The score they gave that outcome in earlier rounds
2. The overall scores given to that outcome by each stakeholder group including their own

All outcomes from round 1 will be retained for subsequent rounds. The project management group will discuss any additional outcomes proposed by participants in round 1 and decide whether the outcome is included within existing outcomes or should be added as a new outcome for round 2.

Attrition between rounds

Although the importance of completion of both rounds of the Delphi survey will be stressed to participants before commencing round 1, it is anticipated that some participants will drop out after each round. Each participant will be ascribed a unique participant number when they sign up to complete round 1 enabling the identification of the attrition rate between rounds. This will allow the identification of participants who have completed both rounds, and analysis of whether participants who drop out before completion of round 2 appear to have views that are different to those who complete the process.

Results and analysis

For peer review only - http://bmjopen.bmj.com/site/about/guidelines.xhtml

Definition of Consensus

A clear definition of what constitutes consensus is essential to reduce potential bias in the interpretation of the results in favour of the opinions of the researchers. Consensus can be considered to have been reached if the majority of participants rank an outcome similarly. After the final round, for each stakeholder group we will assign each outcome to one of three categories:

1. *Consensus in*

   70% or more respondents within a stakeholder group rate the outcome as critically important (7-9) AND 15% or fewer rate the outcome as limited importance (1-3)

2. *Consensus out*

   70% or more of respondents within a stakeholder group rate the outcome as limited importance AND 15% or fewer rate the outcome as critically important (7-9).

3. *No consensus*

   Neither of the above criteria are met.

WP4: Consensus meetings

Research Question

Can we ratify a COS for trials in patients with ASCC through a process which involves all stakeholders?

Overview

The results of the Delphi process will be discussed at a face to face consensus meeting involving an invited sample of Delphi participants from all stakeholder groups. Representatives from secondary stakeholder groups (intended users of the COS including non-clinician trialists; users of the information generated from use of the COS including policy makers guideline developers) will be invited at this stage, in line with the findings of the COMET Minimum Standards in COS development project (Paula Williamson, personal communication). At the meeting, we will propose that any outcome categorised as 'consensus in' across all stakeholder groups be included in the final core outcome set and

For peer review only - http://bmjopen.bmj.com/site/about/guidelines.xhtml

any outcome categorised as ‘consensus out’ across all stakeholder groups be excluded. Attendees will electronically vote to accept this proposal or suggest outcomes from this group that warrant further discussion. All other outcomes, including those categorised as ‘consensus in’ or ‘consensus out’ by 1 or 2 stakeholder groups, and those categorised as ‘no-consensus’ will then be discussed and further rounds of voting will be used to agree the final core outcome set. If a final COS is not agreed at the end of the first consensus meeting, subsequent meetings will be considered.

Recruitment and consent

All participants registering to complete the Delphi process will be additionally offered participation in the consensus meetings (tick box on registration page for Delphi). A sample of participants from both stakeholder groups (patients and HCPs), who have indicated yes to this question and that have completed all rounds of the Delphi process, will be invited to attend the consensus meetings. On the day of the meeting, and prior to commencement of the meeting, patient participants will be asked to confirm their agreement to participate verbally and sign a written consent form.

ETHICS AND DISSEMINATION

Research ethics committee approval for the interviews in WP2 was granted on 22nd December 2015 by the Greater Manchester East research ethics committee. REC reference 15/NW/0971. Research ethics committee approval for the Delphi process in WP3 was granted on 2nd December 2016 by the North East – Newcastle & North Tyneside research ethics committee. REC reference 16/NE/0392. HRA approval was granted on 23rd December 2016.

The benefits of COS are increasingly recognised by research funding bodies, regulators and journal editors, via the work of the COMET Initiative in promoting COS utilisation. The European Medicines Agency recommends COS use for clinical in asthma medicines [23], and the UK National Institute for Health Research (NIHR) mandates outcomes from established COS are included in any new trial proposal [24].

For peer review only - http://bmjopen.bmj.com/site/about/guidelines.xhtml

The robust methodology we have proposed for the development of this COS ensures that health care professionals, trialists and patients are involved at each stage of development. As a whole, the project is overseen by an advisory group including expert representatives from each of these stakeholder groups. This approach will ensure that outcomes in the final core set accurately represent the priorities of those stakeholders. Additionally, the results from the patient interviews undertaken in WP2 will add substantially to the limited body of published literature available on long-term treatment toxicity following pelvic radiotherapy in patients with anal cancer.

Once the final COS is agreed, additional work is planned to develop a core outcome measurement instrument set, in which a single definition or measurement instrument is recommended for each outcome in the COS. Data gathered in the systematic review undertaken in WP1 will allow identification of existing measurement instruments. Identification of instruments will be followed by an assessment and consensus process as described in the COMET/COSMIN 2016 guideline [25].

The output from this project will feed directly into the PLATO (Personalising Anal cancer radiotherapy dOse) anal cancer trials currently in roll-out [26], and into the Association of Coloproctology of Great Britain and Ireland supported national anal cancer audit database. Adoption of the CORMAC COS will help to reduce outcome heterogeneity and therefore increase the quality of information available to health care professionals and patients on which to base informed decisions about treatment.

REFERENCES

1 Wilkinson JR, Morris EJA, Downing A, et al. The rising incidence of anal cancer in England 1990-2010: a population-based study. *Colorectal Disease* 2014;**16**:O234-O9.

2 Islami F, Ferlay J, Lortet-Tieulent J, et al. International trends in anal cancer incidence rates. *International Journal of Epidemiology* 2016:1-15.

3 Glynne-Jones R, Adams R, Lopes A, et al. Clinical endpoints in trials of chemoradiation for patients with anal cancer. *The Lancet Oncology*;**18**:e218-e27.

4 Party UACTW, others. Epidermoid anal cancer: results from the UKCCCR randomised trial of radiotherapy alone versus radiotherapy, 5-fluorouracil, and mitomycin. *The Lancet* 1996;**348**:1049–54.

For peer review only - http://bmjopen.bmj.com/site/about/guidelines.xhtml

5 Bartelink H, Roelofsen F, Eschwege F, et al. Concomitant radiotherapy and chemotherapy is superior to radiotherapy alone in the treatment of locally advanced anal cancer: results of a phase III randomized trial of the European Organization for Research and Treatment of Cancer Radiotherapy and Gastrointestinal Cooperative Groups. *Journal of clinical oncology : official journal of the American Society of Clinical Oncology* 1997;**15**:2040-9.

6 Ajani JA, Winter KA, Gunderson LL, et al. Fluorouracil, mitomycin, and radiotherapy vs fluorouracil, cisplatin, and radiotherapy for carcinoma of the anal canal: a randomized controlled trial. *Jama* 2008;**299**:1914-21.

7 Peiffert D, Tournier-Rangeard L, Gérard J-P, et al. Induction chemotherapy and dose intensification of the radiation boost in locally advanced anal canal carcinoma: final analysis of the randomized UNICANCER ACCORD 03 trial. *Journal of Clinical Oncology* 2012;**30**:1941–8.

8 James RD, Glynne-Jones R, Meadows HM, et al. Mitomycin or cisplatin chemoradiation with or without maintenance chemotherapy for treatment of squamous-cell carcinoma of the anus (ACT II): a randomised, phase 3, open-label, 2 x 2 factorial trial. *Lancet Oncol* 2013;**14**:516-24.

9 Flam M, John M, Pajak TF, et al. Role of mitomycin in combination with fluorouracil and radiotherapy, and of salvage chemoradiation in the definitive nonsurgical treatment of epidermoid carcinoma of the anal canal: results of a phase III randomized intergroup study. *Journal of Clinical Oncology* 1996;**14**:2527-39.

10 Dwan K, Altman DG, Arnaiz JA, et al. Systematic review of the empirical evidence of study publication bias and outcome reporting bias. *PLoS One* 2008;**3**:e3081.

11 Kirkham JJ, Dwan KM, Altman DG, et al. The impact of outcome reporting bias in randomised controlled trials on a cohort of systematic reviews. *BMJ* 2010;**340**:c365.

12 Chan AW, Altman DG. Identifying outcome reporting bias in randomised trials on PubMed: review of publications and survey of authors. *BMJ* 2005;**330**:753.

13 Saini P, Loke YK, Gamble C, et al. Selective reporting bias of harm outcomes within studies: findings from a cohort of systematic reviews. *BMJ* 2014;**349**:g6501.

14 Glynne-Jones R, Renehan A. Current treatment of anal squamous cell carcinoma. *Hematology/oncology clinics of North America* 2012;**26**:1315-50.

15 Boers M, Kirwan JR, Tugwell P, et al. The OMERACT Handbook. *Published by OMERACT(OMERACT)* 2014:52.

16 Williamson PR, Altman DG, Blazeby JM, et al. Developing core outcome sets for clinical trials: issues to consider. *Trials* 2012;**13**:1–8.

17 Machalek DA, Poynten M, Jin F, et al. Anal human papillomavirus infection and associated neoplastic lesions in men who have sex with men: a systematic review and meta-analysis. *The Lancet Oncology* 2012;**13**:487-500.

18 Lee RM. *Doing Research on Sensitive Topics*. SAGE 1993.

19 Francis JJ, Johnston M, Robertson C, et al. What is an adequate sample size? Operationalising data saturation for theory-based interview studies. *Psychology & Health* 2010;**25**:1229-45.

20 Ritchie J, Spencer L. Qualitative data analysis for applied policy research. *Analysing qualitative data*: Taylor and Francis:173-94.

21 Williams PL, Webb C. The Delphi technique: a methodological discussion. *Journal of Advanced Nursing* 1994;**19**:180-6.

22 Guyatt GH, Oxman AD, Vist GE, et al. GRADE: an emerging consensus on rating quality of evidence and strength of recommendations. *Bmj* 2008;**336**:924–6.

For peer review only - http://bmjopen.bmj.com/site/about/guidelines.xhtml

For peer review only

23 Agency EM, Use CfMPfH. Guideline on the clinical investigation of medicinal products for the treatment of ashtma CHMP/EWP/2922/01 Rev.1. European Medicines Agency 2016.
24 Research NIfH. NIHR HTA programme: Guidance notes for completing full proposals. National Institute for Health Research 2016.
25 Prinsen CAC, Vohra S, Rose MR, et al. How to select outcome measurement instruments for outcomes included in a "Core Outcome Set" – a practical guideline. *Trials* 2016;**17**:449.
26 Dewas CV, Maingon P, Dalban C, et al. Does gap-free intensity modulated chemoradiation therapy provide a greater clinical benefit than 3D conformal chemoradiation in patients with anal cancer? *Radiation Oncology* 2012;**7**:201.

SUPPLEMENTARY FILES

Supplementary file 1 is a word document (.doc) of the interview topic guide used in WP2.

DECLARATIONS

Authors contributions

AGR and PW conceived of the project. AGR and PW are the joint principal investigators for the study. RF is the clinical research fellow for the project, is responsible for management of the project and wrote the protocol and manuscript. AGR, PW and CS provide supervision and have had input to all aspects of the project and have commented on drafts of the manuscript. All authors have read and approved the manuscript.

Funding

This paper presents independent research funded by the National Institute for Health Research (NIHR) under its Research for Patient Benefit (RfPB) Programme (Grant Reference Number PB-PG-1013-32064). The views expressed are those of the author(s) and not necessarily those of the NHS, the NIHR or the Department of Health.

For peer review only

Competing Interests

The authors declare they have no competing interests.

List of Abbreviations

ASCC: Anal squamous cell carcinoma; COMET Initiative: Core Outcome Measures in Effectiveness Trials; CORMAC: Core outcome research measures in anal cancer; COS: core outcome set; HIV: Human immunodeficiency virus; HCP: Health care professional; HRA: Health research authority; MSM: Men who have sex with men; NIHR: National Institute for Health Research; OMERACT (Outcomes Measures in Rheumatology); PLATO PersonaLising Anal cancer RadioTherapy dOse. Anal Cancer Trials; REC: Research ethics committee; RfPB: Research for patient benefit; SAG: Study advisory group; WP: work package

For peer review only

INTERVIEW TOPIC GUIDE

Participant No.		Interview location:		Interview date:	
Date of Birth.		Date of diagnsosis		Date of completion of treatment:	
Gender:	Male □<br>Female □		Marital status:	single □<br>married □<br>living with partner □	
HIV status	Positive □<br>Negative □<br>Never tested □		Sexuality	Homosexual □<br>Heterosexual □<br>Bisexual □<br>Prefer not to answer □	
Ethnicity. *(see code sheet)*			Stoma:	Never □<br>Reversed □<br>Temporary □<br>Permanent □	

Introduction:

- Go over the purpose of the study with participant.
- Check they are still willing to take part.
- Check they are happy for interview to be audio recorded.
- Prompt for and answer any other queries.
- **Ask them to fill in the consent form.**

For peer review only

Interview themes

**1)**	Start with a general question about their experience of having anal cancer<br><br>'I understand you have (had) anal cancer. Can you tell me about that?
**2)**	Ask about their experience of being told of their diagnosis<br><br>*'Could you tell me about how you first found out you had anal cancer?'/ 'If I could take you back to when you first learned about your diagnosis?'*<br><br>Prompt for the questions they most wanted to find answers to on being told their diagnosis<br><br>Ask about the treatment that was offered and how they decided about undergoing treatment<br><br>Prompt for what information they wanted about the treatment they would be receiving, and the factors they considered in deciding on a treatment
**3)**	Ask about the treatment that was offered and how they decided about undergoing treatment<br><br>Prompt for what information they wanted about the treatment they would be receiving, and the factors they considered in deciding on a treatment
**4)**	Ask about the effects that treatment had/ is having<br><br>Prompt for specific areas such as physical, mental, effects on relationships<br><br>Prompt about whether they had to modify their behaviour as a result of treatment<br><br>Ask what they considered to be the worst side effect of treatment
**5**	Ask about the long term or permanent side effects of treatment<br><br>Prompt for specific areas such as physical, mental, effects on relationships<br><br>Prompt about whether they had to modify their behaviour as a result of treatment

For peer review only

	Ask what they considered to be the worst side effect of treatment
**6)**	Ask about concerns for the future, especially those relating to their diagnosis/history of anal cancer
**7)**	Ask if there were any areas they wanted more information about but were unable to find<br>Prompt about info leaflets given at time of diagnosis/ treatment
**8)**	Ask whether the explanation of what they should expect from treatment matched their real experience
**9)**	Ask if they can describe what an outcome is in their own words
**10)**	Ask explicitly which outcomes they think it is important to measure
**11)**	Ask whether they think their perspective on what is important has changed over time

For peer review only

STANDARD PROTOCOL ITEMS: RECOMMENDATIONS FOR INTERVENTIONAL TRIALS

SPIRIT 2013 Checklist: Recommended items to address in a clinical trial protocol and related documents*

For peer review only

Section/item	Item No	Description	Addressed on page number
**Administrative information**			
Title	1	Descriptive title identifying the study design, population, interventions, and, if applicable, trial acronym	___1__________
Trial registration	2a	Trial identifier and registry name. If not yet registered, name of intended registry	___3__________
	2b	All items from the World Health Organization Trial Registration Data Set	n/a (not an interventional clinical trial)
Protocol version	3	Date and version identifier	_Footers______
Funding	4	Sources and types of financial, material, and other support	_24__________
Roles and responsibilities	5a	Names, affiliations, and roles of protocol contributors	__1__________
	5b	Name and contact information for the trial sponsor	__3__________
	5c	Role of study sponsor and funders, if any, in study design; collection, management, analysis, and interpretation of data; writing of the report; and the decision to submit the report for publication, including whether they will have ultimate authority over any of these activities	__24_________

For peer review only - http://bmjopen.bmj.com/site/about/guidelines.xhtml

	5d	Composition, roles, and responsibilities of the coordinating centre, steering committee, endpoint adjudication committee, data management team, and other individuals or groups overseeing the trial, if applicable (see Item 21a for data monitoring committee)	__7__________
**Introduction**			
Background and rationale	6a	Description of research question and justification for undertaking the trial, including summary of relevant studies (published and unpublished) examining benefits and harms for each intervention	___5-7________
	6b	Explanation for choice of comparators	n/a (not interventional trial)
Objectives	7	Specific objectives or hypotheses	_5/6__________
Trial design	8	Description of trial design including type of trial (eg, parallel group, crossover, factorial, single group), allocation ratio, and framework (eg, superiority, equivalence, noninferiority, exploratory)	_6-21_______
**Methods: Participants, interventions, and outcomes**			
Study setting	9	Description of study settings (eg, community clinic, academic hospital) and list of countries where data will be collected. Reference to where list of study sites can be obtained	_5___________
Eligibility criteria	10	Inclusion and exclusion criteria for participants. If applicable, eligibility criteria for study centres and individuals who will perform the interventions (eg, surgeons, psychotherapists)	_8; 9; 13-14__
Interventions	11a	Interventions for each group with sufficient detail to allow replication, including how and when they will be administered	__n/a_______
	11b	Criteria for discontinuing or modifying allocated interventions for a given trial participant (eg, drug dose change in response to harms, participant request, or improving/worsening disease)	__ n/a __________
	11c	Strategies to improve adherence to intervention protocols, and any procedures for monitoring adherence (eg, drug tablet return, laboratory tests)	_ n/a _________

For peer review only - http://bmjopen.bmj.com/site/about/guidelines.xhtml

	11d	Relevant concomitant care and interventions that are permitted or prohibited during the trial	__ n/a ________
Outcomes	12	Primary, secondary, and other outcomes, including the specific measurement variable (eg, systolic blood pressure), analysis metric (eg, change from baseline, final value, time to event), method of aggregation (eg, median, proportion), and time point for each outcome. Explanation of the clinical relevance of chosen efficacy and harm outcomes is strongly recommended	__ n/a ________
Participant timeline	13	Time schedule of enrolment, interventions (including any run-ins and washouts), assessments, and visits for participants. A schematic diagram is highly recommended (see Figure)	___ n/a ______
Sample size	14	Estimated number of participants needed to achieve study objectives and how it was determined, including clinical and statistical assumptions supporting any sample size calculations	___ n/a _______
Recruitment	15	Strategies for achieving adequate participant enrolment to reach target sample size	___ n/a ______
**Methods: Assignment of interventions (for controlled trials)**			
Allocation:			
Sequence generation	16a	Method of generating the allocation sequence (eg, computer-generated random numbers), and list of any factors for stratification. To reduce predictability of a random sequence, details of any planned restriction (eg, blocking) should be provided in a separate document that is unavailable to those who enrol participants or assign interventions	___ n/a _______
Allocation concealment mechanism	16b	Mechanism of implementing the allocation sequence (eg, central telephone; sequentially numbered, opaque, sealed envelopes), describing any steps to conceal the sequence until interventions are assigned	___ n/a ______
Implementation	16c	Who will generate the allocation sequence, who will enrol participants, and who will assign participants to interventions	___ n/a ______
Blinding (masking)	17a	Who will be blinded after assignment to interventions (eg, trial participants, care providers, outcome assessors, data analysts), and how	____ n/a _____
	17b	If blinded, circumstances under which unblinding is permissible, and procedure for revealing a participant's allocated intervention during the trial	___ n/a ______

For peer review only - http://bmjopen.bmj.com/site/about/guidelines.xhtml

Methods: Data collection, management, and analysis

Data collection methods	18a	Plans for assessment and collection of outcome, baseline, and other trial data, including any related processes to promote data quality (eg, duplicate measurements, training of assessors) and a description of study instruments (eg, questionnaires, laboratory tests) along with their reliability and validity, if known. Reference to where data collection forms can be found, if not in the protocol	__8 (systematic review); 12 and additional file 1 (patient interviews); 17 (delphi)
	18b	Plans to promote participant retention and complete follow-up, including list of any outcome data to be collected for participants who discontinue or deviate from intervention protocols	19 (Delphi attrition between rounds)_
Data management	19	Plans for data entry, coding, security, and storage, including any related processes to promote data quality (eg, double data entry; range checks for data values). Reference to where details of data management procedures can be found, if not in the protocol	12 (Data analysis-patient interviews); ___________
Statistical methods	20a	Statistical methods for analysing primary and secondary outcomes. Reference to where other details of the statistical analysis plan can be found, if not in the protocol	_19 (Definition of consensus)__
	20b	Methods for any additional analyses (eg, subgroup and adjusted analyses)	__ n/a _________
	20c	Definition of analysis population relating to protocol non-adherence (eg, as randomised analysis), and any statistical methods to handle missing data (eg, multiple imputation)	_ n/a _________

Methods: Monitoring

Data monitoring	21a	Composition of data monitoring committee (DMC); summary of its role and reporting structure; statement of whether it is independent from the sponsor and competing interests; and reference to where further details about its charter can be found, if not in the protocol. Alternatively, an explanation of why a DMC is not needed	__ n/a ______
	21b	Description of any interim analyses and stopping guidelines, including who will have access to these interim results and make the final decision to terminate the trial	__ n/a _____
Harms	22	Plans for collecting, assessing, reporting, and managing solicited and spontaneously reported adverse events and other unintended effects of trial interventions or trial conduct	__ n/a ______

For peer review only - http://bmjopen.bmj.com/site/about/guidelines.xhtml

Auditing	23	Frequency and procedures for auditing trial conduct, if any, and whether the process will be independent from investigators and the sponsor	__ n/a _____
**Ethics and dissemination**			
Research ethics approval	24	Plans for seeking research ethics committee/institutional review board (REC/IRB) approval	__21_________
Protocol amendments	25	Plans for communicating important protocol modifications (eg, changes to eligibility criteria, outcomes, analyses) to relevant parties (eg, investigators, REC/IRBs, trial participants, trial registries, journals, regulators)	_ n/a ________
Consent or assent	26a	Who will obtain informed consent or assent from potential trial participants or authorised surrogates, and how (see Item 32)	_11 & 17___
	26b	Additional consent provisions for collection and use of participant data and biological specimens in ancillary studies, if applicable	_ n/a _______
Confidentiality	27	How personal information about potential and enrolled participants will be collected, shared, and maintained in order to protect confidentiality before, during, and after the trial	_12 (data analysis-patient interviews)___
Declaration of interests	28	Financial and other competing interests for principal investigators for the overall trial and each study site	_24_________
Access to data	29	Statement of who will have access to the final trial dataset, and disclosure of contractual agreements that limit such access for investigators	_24__________
Ancillary and post-trial care	30	Provisions, if any, for ancillary and post-trial care, and for compensation to those who suffer harm from trial participation	_ n/a ______
Dissemination policy	31a	Plans for investigators and sponsor to communicate trial results to participants, healthcare professionals, the public, and other relevant groups (eg, via publication, reporting in results databases, or other data sharing arrangements), including any publication restrictions	__21-22______
	31b	Authorship eligibility guidelines and any intended use of professional writers	__ n/a ________
	31c	Plans, if any, for granting public access to the full protocol, participant-level dataset, and statistical code	__ n/a ________

For peer review only - http://bmjopen.bmj.com/site/about/guidelines.xhtml

Appendices

Informed consent materials	32	Model consent form and other related documentation given to participants and authorised surrogates	available on request- journal guidance does not specify consent forms to be included in submission____________
Biological specimens	33	Plans for collection, laboratory evaluation, and storage of biological specimens for genetic or molecular analysis in the current trial and for future use in ancillary studies, if applicable	__ n/a _______

*It is strongly recommended that this checklist be read in conjunction with the SPIRIT 2013 Explanation & Elaboration for important clarification on the items. Amendments to the protocol should be tracked and dated. The SPIRIT checklist is copyrighted by the SPIRIT Group under the Creative Commons "Attribution-NonCommercial-NoDerivs 3.0 Unported" license.

For peer review only

BMJ Open

Core Outcome Research Measures in Anal Cancer (CORMAC): protocol for systematic review, qualitative interviews and Delphi survey to develop a core outcome set in anal cancer

For peer review only

Journal:	*BMJ Open*
Manuscript ID	bmjopen-2017-018726.R1
Article Type:	Protocol
Date Submitted by the Author:	20-Jul-2017
Complete List of Authors:	Fish, Rebecca; University of Manchester, Division of Cancer Sciences; The Christie NHS Foundation Trust, Colorectal and Peritoneal Oncology Centre<br>Sanders, Caroline; University of Manchester, Centre for Primary Care<br>Williamson, Paula; University of Liverpool, Department of Biostatistics; Medical Research Council, North West Hub for Trials Methodology Research<br>Renehan, Andrew; University of Manchester, Division of Cancer Sciences; The Christie NHS Foundation Trust, Colorectal and Peritoneal Oncology Centre
**Primary Subject Heading**:	Oncology
Secondary Subject Heading:	Evidence based practice, Health policy, Patient-centred medicine, Qualitative research, Surgery
Keywords:	Core Outcome Set, STATISTICS & RESEARCH METHODS, Anal cancer, Patient reported outcomes, Delphi, RADIOTHERAPY

SCHOLARONE™
Manuscripts

Prepared for submission to BMJ Open July 2017

Core Outcome Research Measures in Anal Cancer (CORMAC): protocol for systematic review, qualitative interviews and Delphi survey to develop a core outcome set in anal cancer

Ms Rebecca Fish[1]; Dr Caroline Sanders[2]; Professor Paula Williamson[3]; Professor Andrew G. Renehan[1,4]

1. Clinical Research Fellow, Division of Cancer Sciences, School of Medical Sciences Faculty of Biology, Medicine and Health, University of Manchester, Vaughan House, Portsmouth Street, Manchester, M13 9GB.
rebecca.fish-2@manchester.ac.uk

2. Senior Lecturer in Medical Sociology, Centre for Primary Care, University of Manchester, Williamson Building, 6th Floor, Suite 3, Manchester, M13 9PL.
Caroline.sanders@manchester.ac.uk

3. Professor of Medical Statistics, Department of Biostatistics, Block F Waterhouse Building, University of Liverpool, 1-5 Brownlow Street, Liverpool, L69 3GL.
P.R.Williamson@liverpool.ac.uk

4. Professor in Cancer Studies and Surgery, Honorary Consultant Peritoneal and Colorectal Cancer Surgeon Peritoneal and Colorectal Oncology Centre, Christie NHS Foundation Trust, 550 Wilmslow Road, Manchester, M20 4BX.
andrew.renehan@ics.manchester.ac.uk

Correspondence to:

Dr Rebecca Fish
Clinical Research Fellow,
Division of Cancer Sciences, School of Medical Sciences
Faculty of Biology, Medicine and Health,
University of Manchester,
Vaughan House, Portsmouth Street,
Manchester, M13 9GB.
E-mail: rebecca.fish-2@manchester.ac.uk

Abstract: 299 words; main text: 3994 words; 1 table; 26 references; 1 supplementary file; language: UK English.

For peer review only

ABSTRACT

Introduction

The incidence of anal squamous cell carcinoma (ASCC) has increased 3-fold in the last 30 years. Initial treatment is chemoradiotherapy, associated with short and long-term side effects. Future therapy innovations aim to reduce morbidity in treatment of early tumours whilst maintaining treatment efficacy, and to escalate treatment intensity in locally advanced tumours with acceptable quality of life (QoL). However, all phase III randomised controlled trials to-date have utilised different primary outcomes, which hinders evidence synthesis and presents challenges to the selection of optimal outcomes in future trials. No trial comprehensively assessed long-term side effects and QoL, suggesting outcomes reflecting issues important to patients are under-represented. This project aims to determine the priority outcomes for all stakeholders and reach agreement on a standardized core set of outcomes to be measured and reported on in all future ASCC trials.

Methods & Analysis

A systematic review will identify all outcomes reported in trials and observational studies of chemoradiotherapy as primary treatment for ASCC. Outcomes of importance to patients will be identified through patient interviews. The long list of outcomes generated from the systematic review and interviews will be used to create a two round Delphi process including key stakeholders (patients, health care professionals). The results of the Delphi will be discussed at a face-to-face consensus meeting. Discussion will focus on outcomes that did not achieve consensus through the Delphi process and conclude with anonymous voting to ratify the final core outcome set (COS).

Ethics and Dissemination

The final COS will feed directly into the PLATO national anal cancer trials and the ACPGBI supported national anal cancer database. Utilisation of the COS will increase the relevance of research output to all stakeholders and increase the capacity for data synthesis between trials. This study has ethical approval and is registered with the COMET initiative.

For peer review only - http://bmjopen.bmj.com/site/about/guidelines.xhtml

STUDY REGISTRATION

The study is registered with COMET and listed in their online database.

http://www.comet-initiative.org/studies/details/781

Systematic review:

PROSPERO registration ID: CPMS20368

Phase 1 (semi-structured interviews)

IRAS ID 183034

CPMS study ID; adopted January 2016

Phase 2 (Delphi)

IRAS ID 215791

CPMS Study ID: 33052; adopted February 2017

SPONSOR

The University of Manchester, Christie Building, Oxford Road, Manchester M13 9PL

fbmhethics@manchester.ac.uk.

For peer review only

For peer review only - http://bmjopen.bmj.com/site/about/guidelines.xhtml

Prepared for submission to BMJ Open July 2017

STRENGTHS AND LIMITATIONS OF THIS STUDY

- A core outcome set will facilitate evidence synthesis in anal cancer and ensure future trials utilize outcomes that are relevant to all stakeholders.
- A comprehensive systematic review will identify all outcomes reported in existing trials and observational studies
- Semi-structured interviews with patients will ensure that outcomes that are important to patients are identified
- The consensus phase, constituting a Delphi process and face-to-face consensus meeting, includes international professional and patient participation.
- This project will determine which outcomes to measure, but further work will be necessary to agree and recommend a single measurement instrument or definition for each of the the outcomes in the core outcome set.

For peer review only

For peer review only - http://bmjopen.bmj.com/site/about/guidelines.xhtml

Prepared for submission to BMJ Open July 2017

INTRODUCTION

Anal squamous cell carcinoma (ASCC) is no longer an uncommon malignancy. Since the mid-1980s, incidence rates have increased 3-fold in the UK [1] with 1247 new cases registered in England in 2012 (approximately 1.5 per 100,000 population). Incidence rates are also increasing in other European populations and the in the United States [2]. Treatment of anal cancer is complex. Initial treatment is chemo-radiotherapy, but radical salvage surgery is considered for local relapse which occurs in approximately 20% of cases. Overall, treatment is associated with considerable short- and long-term side effects. Five-year crude survival is approximately 55%, therefore there are many long-term survivors living with treatment-related side effects. Future therapy innovations aim to reduce morbidity in early tumours yet maintain treatment efficacy, whilst escalating treatment intensity with acceptable quality of life (QoL) in locally advanced tumours.

There have been six phase III randomized trials and multiple observational studies of interventions for primary treatment in ASCC, which provide the evidence base for current clinical practice guidelines. Each phase III trial reported different primary outcomes [3] including local failure rate [4]; loco-regional recurrence rate [5]; disease-free survival [6]; colostomy-free survival [7]; complete response [8] and complete pathological response [9]. Furthermore, no trial to-date comprehensively assessed long-term side effects and QoL, suggesting outcomes reflecting issues that may be important to patients are under-represented.

Outcome reporting bias generated by selective reporting of subsets of measured outcome variables is demonstrated in up to 62% of published studies [10] and affects the conclusions in systematic reviews [11]. Outcomes reaching statistical significance have higher odds of being reported compared to non-significant outcomes [12], and harms outcomes reporting is particularly deficient [13].

Outcome heterogeneity and reporting bias reduce the potential for evidence synthesis, which combined with the narrow scope of reported outcomes presents a significant obstacle to

For peer review only - http://bmjopen.bmj.com/site/about/guidelines.xhtml

providing health care professionals and patients with meaningful information on which to base decisions about treatment. Both these issues may be addressed through the development and use of an agreed standardized collection of outcomes, known as a core outcome set (COS), which should be measured and reported, as a minimum, in all studies and trials for a specific clinical area [14]. Currently, there is no COS for trials of treatment in patients with ASCC.

There is no agreed gold standard method for COS development. The COMET Initiative (Core Outcome Measures in Effectiveness Trials) is an organization which aims to facilitate and promote development and use of core outcome sets [14]. They recommend that COS development utilises rigorous consensus methods which involve all stakeholders, including patients, an approach also advocated by the OMERACT (Outcome measures in Rheumatology) group [15]. Here, we will develop a COS for trials in patients with ASCC utilising a recognised stepwise process of information gathering followed by consensus techniques involving all key stakeholder groups.

Aim

The aim of the project is to determine the priority outcomes for all stakeholders and reach agreement on a standardized COS to be measured and reported in all future trials in patients with ASCC.

Scope

The scope of the core outcome set to be developed has been defined according to the criteria recommended by COMET [16].

Health condition: Squamous cell carcinoma of the anus/ anal canal (ASCC)

Population: Adults >18 years of age

Types of Interventions: Primary treatment with radiotherapy with or without concurrent chemotherapy

Setting: Later phase trials that will inform clinical decision making

For peer review only - http://bmjopen.bmj.com/site/about/guidelines.xhtml

METHODS AND ANALYSIS

Taking into consideration the work of OMERACT and COMET, we selected a mixed methods approach for COS development. Development will involve four packages of work over two phases. Phase 1 comprises information gathering, employing literature review and qualitative methods of patient consultation. Phase 2 comprises a process of consolidation and consensus employing a Delphi process and structured group discussion involving all stakeholder groups.

Project oversight

A study advisory group (SAG) has been assembled to oversee the project. Members include oncologists with leading roles in past and current anal cancer clinical trials, a colorectal surgeon, an anal cancer specialist nurse, a COS methodological expert, a qualitative methodology expert and a patient representative.

Phase 1: Information gathering

The aim of the information gathering stage is to generate a comprehensive list of all outcomes relating to the initial treatment of patients with ASCC using chemoradiotherapy. The primary list will be generated by extracting outcomes from the published literature on the subject through a systematic review (WP1). The published literature will be assumed to represent the views of health care professionals and trialists. The primary list will be supplemented with any additional outcomes that are identified through a series of semi-structured interviews with individuals who have, or have had anal cancer (WP2).

For peer review only - http://bmjopen.bmj.com/site/about/guidelines.xhtml

WP1: Systematic review

Research question

Which outcomes are in use in the published literature on initial treatment of patients with ASCC using radiotherapy with or without concurrent chemotherapy?

Method

A systematic review of the literature will be performed to identify a comprehensive list of all outcomes in use in trials and observational studies in patients with ASCC undergoing initial treatments. The full protocol, including search strategy and study selection criteria, is available online via the PROSPERO database [17].

WP2: Patient consultation

Research Question

What are the outcomes patients with anal cancer regard as potentially important following treatment?

Methods

Inclusion criteria

Types of participant

- Adults > 18 years of age.
- Patients who have completed or are receiving initial treatment for ASCC
- Able to participate in an interview in the English language

Types of pathology

- Anal canal or anal canal and margin cancer of the following histological sub-types that collectively make up the entity of ASCC: squamous cell, basaloid, baso-squamous, cloacogenic and transitional cell tumours.

For peer review only - http://bmjopen.bmj.com/site/about/guidelines.xhtml

Types of Intervention

- External (non-contact) radiotherapy with or without concurrent chemotherapy as initial treatment with curative intent for anal cancer.

Exclusion criteria

Types of participant

- Unable to give informed consent
- Too unwell to comfortably participate in an interview lasting approximately 30-60 minutes. Types of pathology

Types of pathology

- Anal intra-epithelial neoplasia (AIN) only
- Anal tumours of histological type other than SCC, including adenocarcinoma, melanoma and other rare tumours

Types of intervention

- Treatment for anal cancer with purely palliative intent
- Salvage surgery for anal cancer following primary chemo-radiotherapy
- Any non-radiotherapy initial treatment for anal cancer

Sampling

The majority of participants will be purposively drawn from the prospectively maintained database of patients with anal cancer at The Christie NHS Foundation Trust. We will utilise existing networks of cancer survivors via national support groups in order to invite participants. A purposive approach to sampling has been selected with the aim of maximising diversity within the study participants. Criteria have been selected to ensure that subsets within the study population that may express contrasting views and experiences are represented. These criteria for difference will be used to populate a sampling matrix (Table 1).

Table 1 Sampling matrix for purposing sampling of participants in WP2.

For peer review only - http://bmjopen.bmj.com/site/about/guidelines.xhtml

Key Criteria	Target Number of participants
**Age at diagnosis**	
18-30	3-4
31-65	10-12
65+	3-4
**Treatment stage**	
Undergoing primary treatment	5-7
Completed primary treatment <5 years ago	5-7
Completed primary treatment >5 years ago	5-7
**Stoma**	
Current stoma or previous stoma	2-4
**Gender**	
Male	6-8
Female	6-8
**Sexuality**	
MSM	2-4
**HIV status**	
HIV positive	2-4
**Target total**	**20**

MSM: Men who have sex with men *Note: The 'Target Total' refers to the total number of participants but it is not the sum of the individual criteria because many participants will fall into several categories e.g. a male patient with a stoma who completed treatment >5 years ago.*

The key criteria for identifying difference will include:

- Age at diagnosis
- Treatment stage
- HIV status
- Sexuality (specifically men who have sex with men or MSM)
- Gender

In order to ensure inclusion of minority groups which can be hard to reach (e.g. MSM), snowball sampling will be used by asking participants to suggest contacts known to them

For peer review only - http://bmjopen.bmj.com/site/about/guidelines.xhtml

who may be willing to participate. This is a common technique used for researching sensitive topics and for gaining access to hard to reach populations [18].

Sample Size

We will conduct up to 30 interviews and final sample size will be contingent on iterative analysis to achieve 'saturation' in terms of identifying recurring themes in analysis of the data as described by Francis [19].

Consent

Individuals who do not have capacity to give informed consent will not be included in the study, and any participant who is deemed to have lost capacity to give consent during the study will be withdrawn from the study. Information for potential participants will be provided verbally and in the approved information sheets. It will be stressed that the individual is under no obligation to take part and they are free to withdraw at any time without affecting their medical care.

Interview location

Interviews will take place at a time and place convenient to the participant. Choices of location with include:

1. A clinic room at the Christie NHS Foundation Trust
2. A room at the University of Manchester
3. The participant's home
4. Via telephone

Participants will be reimbursed for travel expenses for travelling to and from interview locations.

Interview format

For peer review only

For peer review only - http://bmjopen.bmj.com/site/about/guidelines.xhtml

Interviews will explore patients' perceptions, priorities and experiences of living with and having treatment for anal cancer, using a semi-structured format. This approach uses open questions to facilitate a patient-led discussion, guided by additional prompts from a pre-prepared topic guide to ensure key areas are covered [see supplementary file 1]. The topic guide may be modified iteratively during the series of patient interviews to ensure inclusion of items that have been raised by earlier participants but not included in the topic guide are covered in subsequent discussions.

Data Analysis

Interviews will be audio-recorded and transcribed in full. Transcription will be performed by an approved secretarial service. Data will be analysed through thematic analysis by the Framework method [20] using NVivo 10 software. The data will be indexed and charted to produce a matrix of themes and cases and these will be discussed and agreed by multiple members of the research team (RF & CS). Themes will be derived from issues raised by participants. From this analysis, we will develop a list of outcomes of key importance to survivors of anal cancer. Only members of the project management group will have access to transcripts.

Phase 2: Consolidation and Consensus

A meeting of the study advisory group will be held to discuss and agree on a comprehensive list of outcomes identified from the patient interviews and systematic review. Discussion of the identified outcomes will ensure clear and efficient meanings are given, and that there is no duplication. The long list created from this meeting will be used to create the Delphi survey used in WP3.

WP3: Delphi process

A process of iterative surveys (Delphi process) will be undertaken involving the two key stakeholder groups (patients and health care professionals including clinician trialists)

For peer review only - http://bmjopen.bmj.com/site/about/guidelines.xhtml

adhering to the standards recommended by the COMET Minimum Standards In COS Development project (Paula Williamson, personal communication). Questionnaires are administered in 2 sequential rounds, with anonymised feedback of the results of the previous round provided to participants before completion of the subsequent round. This process is intended to achieve consensus amongst participants by minimising the potential for bias towards the opinions of those who are more outspoken or whose views might be perceived as superior. The aim of the Delphi process is to move towards consensus amongst stakeholders over which outcomes from the long list generated in phase 1 should be considered for inclusion in the final COS.

Research question

Which outcomes do patients and health care professionals think should be included in a core outcome set for trials of patients with ASCC?

Method

Participants

Participants will be recruited from the two key stakeholder groups: patients and health care professionals (HCPs). Clinicians involved in clinical trials will form a subgroup within the HCP stakeholder group.

Inclusion criteria

All participants must be adults > 18 years of age and able to complete a questionnaire in the English language

Patients

- Patients who have completed or are receiving initial treatment using chemoradiotherapy for ASCC.

Health care professionals

For peer review only - http://bmjopen.bmj.com/site/about/guidelines.xhtml

All members of the clinical team involved in the management of individuals who have or have had anal cancer, including all members of the MRT are eligible to participate. This will include:

- Clinical oncologists
- Radiologists
- Radiographers
- Pathologists
- Specialist nurses
- Colorectal surgeons
- Stoma nurses
- Gastroenterologists
- Radio-physicists

Sampling

Patients

All UK centres offering radiotherapy based treatment for patients with ASCC will be invited to become participant identification centres (PICs). Each PIC will be asked to nominate a member of the clinical team (likely a research nurse or clinical nurse specialist) to identify potential patient participants from clinic lists or patient records. They will then distribute recruitment letters to the identified individuals, either in person during routine follow-up visits, or by post. The recruitment letter will give a full explanation of the Delphi process and instructions of how to contact the research team for more information by e-mail, phone or post. We will ask PICs to display posters advertising the study in appropriate waiting rooms and patient areas. Potential participants contacting the research team will be given details of how to register to take part. The importance of completing all rounds of the Delphi process will be stressed at this stage to try and minimise inter-round attrition.

For peer review only - http://bmjopen.bmj.com/site/about/guidelines.xhtml

Links have been established with a number of patient groups internationally (for example, HPV and Anal Cancer Foundation, Pelvic Radiation Disease Association). A named contact at the group will act as a liaison member and will circulate to other members the promotional poster and contact details for the research team. Recruitment posters and e-mail contact for the research team will be disseminated via patient support group websites and via social media sites including twitter.

Health Care Professionals

All members of each UK regional anal cancer MDT will be contacted and invited to participate.

The membership of international associations and/or their disease relevant subgroups will be contacted and invited to participate:

- Association of Coloproctology of Great Britain and Ireland
- European Society of Coloproctology
- European Society for Radiotherapy and Oncology
- American Society of Colon and Rectal Surgeons
- American Society for Radiation Oncology
- Nordic Anal Cancer Group
- Colorectal surgical society of Australia and New Zealand
- Trans-Tasman Radiation Oncology Group

Contacts of the CORMAC study advisory group will be contacted and invited to participate.

Snowball sampling will be allowed to increase sample size.

Trialists

Corresponding authors of the following will be contacted and invited to participate:

- The 6 phase III randomised trials in anal cancer
- The working group developing the protocol for the planned international PLATO anal cancer trial

For peer review only - http://bmjopen.bmj.com/site/about/guidelines.xhtml

For peer review only

- Large cohort studies and non-randomised trials published in the last 2 years.
- International Rare Cancers Group

Recruitment

Potential participants will be contacted either by e-mail or post. Correspondence will outline the rationale for the development of a core outcome set and describe the requirements for taking part in the Delphi. In particular, the importance of completing all rounds of the questionnaire will be emphasised in an effort to reduce inter-round drop-out.

All participants will be invited to pass on details or the study to any of their own contacts who meet the eligibility requirements (snowball sampling) to increase sample size and reach.

Sample size

There are no recommendations for the number of participants to include in a Delphi survey [21]. We will therefore take a pragmatic approach to sample size and aim to invite all individuals who meet the inclusion criteria as identified by the approach set out above. We will keep a record of the source of all participants and record the number of invited and the number recruited for each stakeholder group. No new participants will be invited after commencement of the round 1 questionnaire.

Consent

No explicit consent will be taken for completion of the questionnaire. Consent will be implicit by the process of registering to take part in the Delphi process via the website and by completion and return of questionnaires. It will be clearly stated on the Delphi registration page that registering to participate by submitting their name and e-mail address is indicating their agreement to participate in the Delphi process.

Questionnaires

For peer review only - http://bmjopen.bmj.com/site/about/guidelines.xhtml

The questionnaire will be built and administered in an online format using the DelphiManager software developed by the COMET group. Participants will be asked to select which of the stakeholder groups (patient; HCP) they belong to prior to commencing the questionnaire. Further information specific to each stakeholder group will then be gathered:

Patients

- Age
- Months since completion of treatment
- Gender
- Sexuality
- Ethnicity
- Country in which received treatment for ASCC

Health care professionals

- Discipline (medical oncologist, specialist nurse etc)
- Involvement with trials (named author on publication of a trial of chemoradiotherapy in anal cancer; part of working group involved in a trial of chemoradiotherapy in anal cancer; part of working group for development of future trials in anal cancer
- Country of practice

Instructions for how to complete the questionnaire will be included at the start of each round. Participants will be asked to rate the importance of each outcome based the scale proposed by the GRADE working group [22]. This is a 9 point Likert scale, grouped into 3 categories: 1-3 (limited importance); 4-6 (important but not critical) and 7-9 (critically important).

Within the questionnaire outcomes will be grouped into domains so that similar or related outcomes are viewed together. Each outcome will be described in medical terms and in plain language, with participants able to toggle between versions. The language used will be

For peer review only - http://bmjopen.bmj.com/site/about/guidelines.xhtml

piloted on patients and health care professionals prior to finalising the questionnaire to ensure clarity and consistency of meaning.

Participants will be able to suggest additional outcomes to include in subsequent rounds.

Delphi rounds and feedback

Two rounds of the Delphi questionnaire will be undertaken. The spread of scores for each question item should be seen to reduce from round 1 to round 2 as consensus is reached (see definition of consensus in next section).

For all rounds after the first round, participants will be able to review the results from earlier rounds as they rate each outcome. Each participant will be able to see:

1. The score they gave that outcome in earlier rounds
2. The overall scores given to that outcome by each stakeholder group including their own

All outcomes from round 1 will be retained for subsequent rounds. The project management group will discuss any additional outcomes proposed by participants in round 1 and decide whether the outcome is included within existing outcomes or should be added as a new outcome for round 2.

Attrition between rounds

Although the importance of completion of both rounds of the Delphi survey will be stressed to participants before commencing round 1, it is anticipated that some participants will drop out after each round. Each participant will be ascribed a unique participant number when they sign up to complete round 1 enabling the identification of the attrition rate between rounds. This will allow the identification of participants who have completed both rounds, and analysis of whether participants who drop out before completion of round 2 appear to have views that are different to those who complete the process.

Results and analysis

For peer review only - http://bmjopen.bmj.com/site/about/guidelines.xhtml

Definition of Consensus

A clear definition of what constitutes consensus is essential to reduce potential bias in the interpretation of the results in favour of the opinions of the researchers. Consensus can be considered to have been reached if the majority of participants rank an outcome similarly. After the final round, for each stakeholder group we will assign each outcome to one of three categories:

1. *Consensus in*

   70% or more respondents within a stakeholder group rate the outcome as critically important (7-9) AND 15% or fewer rate the outcome as limited importance (1-3)

2. *Consensus out*

   70% or more of respondents within a stakeholder group rate the outcome as limited importance AND 15% or fewer rate the outcome as critically important (7-9).

3. *No consensus*

   Neither of the above criteria are met.

WP4: Consensus meetings

Research Question

Can we ratify a COS for trials in patients with ASCC through a process which involves all stakeholders?

Overview

The results of the Delphi process will be discussed at a face to face consensus meeting involving an invited sample of Delphi participants from all stakeholder groups. Representatives from secondary stakeholder groups (intended users of the COS including non-clinician trialists; users of the information generated from use of the COS including policy makers guideline developers) will be invited at this stage, in line with the findings of the COMET Minimum Standards in COS development project (Paula Williamson, personal communication). At the meeting, we will propose that any outcome categorised as ‘consensus in’ across all stakeholder groups be included in the final core outcome set and

For peer review only - http://bmjopen.bmj.com/site/about/guidelines.xhtml

any outcome categorised as 'consensus out' across all stakeholder groups be excluded. Attendees will electronically vote to accept this proposal or suggest outcomes from this group that warrant further discussion. All other outcomes, including those categorised as 'consensus in' or 'consensus out' by 1 or 2 stakeholder groups, and those categorised as 'no-consensus' will then be discussed and further rounds of voting will be used to agree the final core outcome set. If a final COS is not agreed at the end of the first consensus meeting, subsequent meetings will be considered.

Recruitment and consent

All participants registering to complete the Delphi process will be additionally offered participation in the consensus meetings (tick box on registration page for Delphi). A sample of participants from both stakeholder groups (patients and HCPs), who have indicated yes to this question and that have completed all rounds of the Delphi process, will be invited to attend the consensus meetings. On the day of the meeting, and prior to commencement of the meeting, patient participants will be asked to confirm their agreement to participate verbally and sign a written consent form.

ETHICS AND DISSEMINATION

Research ethics committee approval for the interviews in WP2 was granted on 22nd December 2015 by the Greater Manchester East research ethics committee. REC reference 15/NW/0971. Research ethics committee approval for the Delphi process in WP3 was granted on 2nd December 2016 by the North East – Newcastle & North Tyneside research ethics committee. REC reference 16/NE/0392. HRA approval was granted on 23rd December 2016.

The benefits of COS are increasingly recognised by research funding bodies, regulators and journal editors, via the work of the COMET Initiative in promoting COS utilisation. The European Medicines Agency recommends COS use for clinical in asthma medicines [23], and the UK National Institute for Health Research (NIHR) mandates outcomes from established COS are included in any new trial proposal [24].

For peer review only - http://bmjopen.bmj.com/site/about/guidelines.xhtml

The robust methodology we have proposed for the development of this COS ensures that health care professionals, trialists and patients are involved at each stage of development. As a whole, the project is overseen by an advisory group including expert representatives from each of these stakeholder groups. This approach will ensure that outcomes in the final core set accurately represent the priorities of those stakeholders. Additionally, the results from the patient interviews undertaken in WP2 will add substantially to the limited body of published literature available on long-term treatment toxicity following pelvic radiotherapy in patients with anal cancer.

Once the final COS is agreed, additional work is planned to develop a core outcome measurement instrument set, in which a single definition or measurement instrument is recommended for each outcome in the COS. Data gathered in the systematic review undertaken in WP1 will allow identification of existing measurement instruments. Identification of instruments will be followed by an assessment and consensus process as described in the COMET/COSMIN 2016 guideline [25].

The output from this project will feed directly into the PLATO (Personalising Anal cancer radiotherapy dOse) anal cancer trials currently in roll-out [26], and into the Association of Coloproctology of Great Britain and Ireland supported national anal cancer audit database. Adoption of the CORMAC COS will help to reduce outcome heterogeneity and therefore increase the quality of information available to health care professionals and patients on which to base informed decisions about treatment.

REFERENCES

1 Wilkinson JR, Morris EJA, Downing A, et al. The rising incidence of anal cancer in England 1990-2010: a population-based study. *Colorectal Disease* 2014;**16**:O234-O9.
2 Islami F, Ferlay J, Lortet-Tieulent J, et al. International trends in anal cancer incidence rates. *International Journal of Epidemiology* 2016:1-15.
3 Glynne-Jones R, Adams R, Lopes A, et al. Clinical endpoints in trials of chemoradiation for patients with anal cancer. *The Lancet Oncology*;**18**:e218-e27.
4 Party UACTW, others. Epidermoid anal cancer: results from the UKCCCR randomised trial of radiotherapy alone versus radiotherapy, 5-fluorouracil, and mitomycin. *The Lancet* 1996;**348**:1049–54.

For peer review only - http://bmjopen.bmj.com/site/about/guidelines.xhtml

5 Bartelink H, Roelofsen F, Eschwege F, et al. Concomitant radiotherapy and chemotherapy is superior to radiotherapy alone in the treatment of locally advanced anal cancer: results of a phase III randomized trial of the European Organization for Research and Treatment of Cancer Radiotherapy and Gastrointestinal Cooperative Groups. *Journal of clinical oncology : official journal of the American Society of Clinical Oncology* 1997;**15**:2040-9.
6 Ajani JA, Winter KA, Gunderson LL, et al. Fluorouracil, mitomycin, and radiotherapy vs fluorouracil, cisplatin, and radiotherapy for carcinoma of the anal canal: a randomized controlled trial. *Jama* 2008;**299**:1914-21.
7 Peiffert D, Tournier-Rangeard L, Gérard J-P, et al. Induction chemotherapy and dose intensification of the radiation boost in locally advanced anal canal carcinoma: final analysis of the randomized UNICANCER ACCORD 03 trial. *Journal of Clinical Oncology* 2012;**30**:1941–8.
8 James RD, Glynne-Jones R, Meadows HM, et al. Mitomycin or cisplatin chemoradiation with or without maintenance chemotherapy for treatment of squamous-cell carcinoma of the anus (ACT II): a randomised, phase 3, open-label, 2 x 2 factorial trial. *Lancet Oncol* 2013;**14**:516-24.
9 Flam M, John M, Pajak TF, et al. Role of mitomycin in combination with fluorouracil and radiotherapy, and of salvage chemoradiation in the definitive nonsurgical treatment of epidermoid carcinoma of the anal canal: results of a phase III randomized intergroup study. *Journal of Clinical Oncology* 1996;**14**:2527-39.
10 Dwan K, Altman DG, Arnaiz JA, et al. Systematic review of the empirical evidence of study publication bias and outcome reporting bias. *PLoS One* 2008;**3**:e3081.
11 Kirkham JJ, Dwan KM, Altman DG, et al. The impact of outcome reporting bias in randomised controlled trials on a cohort of systematic reviews. *BMJ* 2010;**340**:c365.
12 Chan AW, Altman DG. Identifying outcome reporting bias in randomised trials on PubMed: review of publications and survey of authors. *BMJ* 2005;**330**:753.
13 Saini P, Loke YK, Gamble C, et al. Selective reporting bias of harm outcomes within studies: findings from a cohort of systematic reviews. *BMJ* 2014;**349**:g6501.
14 Glynne-Jones R, Renehan A. Current treatment of anal squamous cell carcinoma. *Hematology/oncology clinics of North America* 2012;**26**:1315-50.
15 Boers M, Kirwan JR, Tugwell P, et al. The OMERACT Handbook. *Published by OMERACT(OMERACT)* 2014:52.
16 Williamson PR, Altman DG, Blazeby JM, et al. Developing core outcome sets for clinical trials: issues to consider. *Trials* 2012;**13**:1–8.
17 Fish R, Williamson PR, Sanders C, et al. CORMAC: Core Outcome Research Measures in Anal Cancer. Protocol for a systematic review of outcomes measured and reported in studies of anal squamous cell carcinoma treated with primary chemoradiotherapy. PROSPERO International prospective register of systematic reviews: PROSPERO 2016..
18 Lee RM. *Doing Research on Sensitive Topics*. SAGE 1993.
19 Francis JJ, Johnston M, Robertson C, et al. What is an adequate sample size? Operationalising data saturation for theory-based interview studies. *Psychology & Health* 2010;**25**:1229-45.
20 Ritchie J, Spencer L. Qualitative data analysis for applied policy research. *Analysing qualitative data*: Taylor and Francis:173-94.
21 Williams PL, Webb C. The Delphi technique: a methodological discussion. *Journal of Advanced Nursing* 1994;**19**:180-6.

For peer review only - http://bmjopen.bmj.com/site/about/guidelines.xhtml

22 Guyatt GH, Oxman AD, Vist GE, et al. GRADE: an emerging consensus on rating quality of evidence and strength of recommendations. *Bmj* 2008;**336**:924–6.
23 Agency EM, Use CfMPfH. Guideline on the clinical investigation of medicinal products for the treatment of ashtma CHMP/EWP/2922/01 Rev.1. European Medicines Agency 2016.
24 Research NIfH. NIHR HTA programme: Guidance notes for completing full proposals. National Institute for Health Research 2016.
25 Prinsen CAC, Vohra S, Rose MR, et al. How to select outcome measurement instruments for outcomes included in a “Core Outcome Set” – a practical guideline. *Trials* 2016;**17**:449.
26 Dewas CV, Maingon P, Dalban C, et al. Does gap-free intensity modulated chemoradiation therapy provide a greater clinical benefit than 3D conformal chemoradiation in patients with anal cancer? *Radiation Oncology* 2012;**7**:201.

SUPPLEMENTARY FILES

Supplementary file 1 is a word document (.doc) of the interview topic guide used in WP2.

DECLARATIONS

Authors contributions

AGR and PW conceived of the project. AGR and PW are the joint principal investigators for the study. RF is the clinical research fellow for the project, is responsible for management of the project and wrote the protocol and manuscript. AGR, PW and CS provide supervision and have had input to all aspects of the project and have commented on drafts of the manuscript. All authors have read and approved the manuscript.

Funding

This paper presents independent research funded by the National Institute for Health Research (NIHR) under its Research for Patient Benefit (RfPB) Programme (Grant Reference Number PB-PG-1013-32064). The views expressed are those of the author(s) and not necessarily those of the NHS, the NIHR or the Department of Health.

For peer review only - http://bmjopen.bmj.com/site/about/guidelines.xhtml

Competing Interests

The authors declare they have no competing interests.

List of Abbreviations

ASCC: Anal squamous cell carcinoma; COMET Initiative: Core Outcome Measures in Effectiveness Trials; CORMAC: Core outcome research measures in anal cancer; COS: core outcome set; HIV: Human immunodeficiency virus; HCP: Health care professional; HRA: Health research authority; MSM: Men who have sex with men; NIHR: National Institute for Health Research; OMERACT (Outcomes Measures in Rheumatology); PLATO PersonaLising Anal cancer RadioTherapy dOse. Anal Cancer Trials; REC: Research ethics committee; RfPB: Research for patient benefit; SAG: Study advisory group; WP: work package

For peer review only

For peer review only - http://bmjopen.bmj.com/site/about/guidelines.xhtml

CORMAC INTERVIEW TOPIC GUIDE

Participant No.		Interview location:		Interview date:	
Date of Birth.		Date of diagnosis		Date of completion of treatment:	
Gender:	Male □<br>Female □		Marital status:	single □<br>married □<br>living with partner □	
HIV status	Positive □<br>Negative □<br>Never tested □		Sexuality	Homosexual □<br>Heterosexual □<br>Bisexual □<br>Prefer not to answer □	
Ethnicity.<br>*(see code sheet)*			Stoma:	Never □<br>Reversed □<br>Temporary □<br>Permanent □	

Introduction:

- Go over the purpose of the study with participant.
- Check they are still willing to take part.
- Check they are happy for interview to be audio recorded.
- Prompt for and answer any other queries.
- **Ask them to fill in the consent form.**

For peer review only

For peer review only - http://bmjopen.bmj.com/site/about/guidelines.xhtml

Interview themes

**1)**	Start with a general question about their experience of having anal cancer<br><br>'I understand you have (had) anal cancer. Can you tell me about that?
**2)**	Ask about their experience of being told of their diagnosis<br><br>*'Could you tell me about how you first found out you had anal cancer?'/ 'If I could take you back to when you first learned about your diagnosis?'*<br><br>Prompt for the questions they most wanted to find answers to on being told their diagnosis<br><br>Ask about the treatment that was offered and how they decided about undergoing treatment<br><br>Prompt for what information they wanted about the treatment they would be receiving, and the factors they considered in deciding on a treatment
**3)**	Ask about the treatment that was offered and how they decided about undergoing treatment<br><br>Prompt for what information they wanted about the treatment they would be receiving, and the factors they considered in deciding on a treatment
**4)**	Ask about the effects that treatment had/ is having<br><br>Prompt for specific areas such as physical, mental, effects on relationships<br><br>Prompt about whether they had to modify their behaviour as a result of treatment<br><br>Ask what they considered to be the worst side effect of treatment
**5**	Ask about the long term or permanent side effects of treatment<br><br>Prompt for specific areas such as physical, mental, effects on relationships<br><br>Prompt about whether they had to modify their behaviour as a result of treatment

For peer review only - http://bmjopen.bmj.com/site/about/guidelines.xhtml

	Ask what they considered to be the worst side effect of treatment
**6)**	Ask about concerns for the future, especially those relating to their diagnosis/history of anal cancer
**7)**	Ask if there were any areas they wanted more information about but were unable to find<br>Prompt about info leaflets given at time of diagnosis/ treatment
**8)**	Ask whether the explanation of what they should expect from treatment matched their real experience
**9)**	Ask if they can describe what an outcome is in their own words
**10)**	Ask explicitly which outcomes they think it is important to measure
**11)**	Ask whether they think their perspective on what is important has changed over time

For peer review only - http://bmjopen.bmj.com/site/about/guidelines.xhtml
For peer review only
