# Supplementary material for: Core outcome research measures in anal cancer (CORMAC): protocol for systematic review, qualitative interviews and Delphi survey to develop a core outcome set in anal cancer
Source: BMJ Open. 2017 Nov 22;7(11):e018726. doi: 10.1136/bmjopen-2017-018726 (PMC5719280; doi:10.1136/bmjopen-2017-018726)
Supplement: Reviewer comments [file bmjopen-2017-018726.reviewer_comments.pdf]

PEER REVIEW HISTORY

BMJ Open publishes all reviews undertaken for accepted manuscripts. Reviewers are asked to complete a checklist review form (**http://bmjopen.bmj.com/site/about/resources/checklist.pdf**) and are provided with free text boxes to elaborate on their assessment. These free text comments are reproduced below.

ARTICLE DETAILS

**TITLE (PROVISIONAL)**	Core Outcome Research Measures in Anal Cancer (CORMAC): protocol for systematic review, qualitative interviews and Delphi survey to develop a core outcome set in anal cancer
**AUTHORS**	Fish, Rebecca; Sanders, Caroline; Williamson, Paula; Renehan, Andrew

This article was not externally reviewed at BMJ Open. Protocols that have been independently assessed prior to submission to BMJ Open are usually fast-tracked to publication on the grounds that further substantial changes will not be possible. This independent assessment will usually be external, independent review for both a substantial grant award from a non-commercial or government funder and ethics approval.
